# Real-Time Dynamic Adaptive Test Assembly for Personalized Measurement of Cognitive Abilities Under Continuous Item Bank Growth

**DOI:** 10.3390/jintelligence14090216

**Published:** 2026-09-06

**Authors:** Jiawei Xiong, Cheng Tang, Qidi Liu, Feiming Li

**Affiliations:** 1 Department of Educational Psychology, Mary Frances Early College of Education, University of Georgia, Athens, GA 30605, USA; jiawei.xiong@uga.edu (J.X.); cheng.tang@uga.edu (C.T.); qidi.liu.photonics@gmail.com (Q.L.); 2 Zhejiang Key Laboratory of Intelligent Education Technology and Application, College of Education, Zhejiang Normal University, Jinhua 321004, China

**Keywords:** personalized assessment, intelligence measurement, cognitive ability estimation, automated test assembly, item parameter prediction, psychometrics, uncertainty quantification, personalized learning, Ising model

## Abstract

The accurate measurement of individual cognitive abilities in educational settings increasingly relies on personalized assessment systems that adapt to the learner’s current standing on the measured ability. These systems generate and use assessment items continuously and in real-time, bypassing the field-test cycle that traditional calibration requires. Item banks therefore contain a growing share of items whose parameters must be predicted first, and each test form must be assembled in real time to match the learner’s current ability level and content needs for effective personalized instruction. This study proposes an Ising framework that addresses both requirements by predicting item parameters from text with calibrated uncertainty estimates and assembling personalized test forms within the time budget of the assessment loop. This framework is validated through a factorial simulation and an empirical study on state-level English Language Arts items across Grades 3 through 12. In the simulation, the proposed uncertainty-aware method achieves a mean test information function (TIF) gap of 0.226, compared with 1.171 for the conventional baseline that ignores prediction uncertainty. The proposed method also produces feasible forms across the full operational region, whereas the chance-constrained and robust baselines become infeasible at high prediction error combined with a high share of predicted items. In the empirical study, predictive intervals achieve coverage within ten percentage points of the nominal level in the pooled condition, and the uncertainty-aware assembly reduces the TIF gap relative to the baseline, with the largest gains at the extremes of the ability distribution where accurate measurement matters most for instructional placement decisions. By separating prediction uncertainty from true individual differences in ability, this framework preserves the interpretability of cognitive ability estimates and provides a foundation for personalized assessment systems that support differentiated instruction under continuous item bank growth.

## 1. Introduction

### 1.1. Personalized Test Assembly

A central goal of intelligence measurement is to characterize each learner’s current ability level accurately enough to guide instructional decisions (X. Xiong et al., 2026). When the measurement of cognitive abilities is personalized in this way, it can inform differentiated instruction that responds to each student’s current level and learning trajectory (J. Xiong & Li, 2023). Assessment of this kind calls for item banks that are diverse in content, that grow continually as new standards and skills are addressed, and that can be matched to each learner in real time. Advances in natural language processing now make this process practical, and there is correspondingly growing interest in automatically assembling assessments from newly generated items whose difficulty is predicted rather than empirically calibrated, particularly in personalized learning environments (von Davier, 2018; Benedetto et al., 2020). For example, continuous testing windows are replacing single administration cycles, item exposure on commercial homework platforms forces the frequent retirement of overused items, and item generation assisted by large language models (LLMs) is now operationally deployed in adaptive language tests given the development of Artificial Intelligence (AI) (Settles et al., 2020). These developments make the accurate prediction of item parameters increasingly important, because newly generated items may need to contribute to ability estimation before sufficient response data are available for conventional calibration.

Accordingly, item parameter prediction has become an increasingly active research topic. However, using predicted rather than empirically calibrated parameters introduces an additional source of uncertainty into ability estimation. An ability estimate is interpretable when the standard error attached to it is trustworthy. When item parameters are predicted from text rather than estimated from response data, the prediction error is absorbed into the ability estimate without appearing in the reported precision. Variation that appears to reflect individual differences in ability may therefore reflect variation in the item parameters instead. Understanding this problem requires examining how errors in predicted item parameters propagate through the psychometric model used to estimate learner ability.

For assessments designed to measure a single ability within a specific grade and content domain, this psychometric framework is typically provided by unidimensional item response theory (IRT). For example, reading and writing ability is represented as a narrow ability in the Cattell–Horn–Carroll taxonomy of cognitive abilities (Carroll, 1993; McGrew, 2009; Tang et al., 2025a, 2025b). Accordingly, a unidimensional two-parameter logistic (2PL) model or three-parameter logistic (3PL) model is appropriate at this level of the hierarchy. In personalized learning environments, however, these models must operate under conditions that differ substantially from those of conventional assessment; for instance, newly generated items may enter the assessment immediately, and ability estimates must be updated repeatedly in real time.

Personalized learning systems can now author assessment items on demand with the assistance of LLMs, and they can administer those items within hours or days of authoring. This creates a rapid assessment cycle in which item generation, item selection, response collection, and ability estimation are tightly coupled. When a student signs in, the system selects a small set of items matched to the student’s current learning standard and estimated ability. After the student responds, the ability estimate is updated, and the system selects the next set of items. Each cycle of this loop must complete within a few seconds, and it must respect the content blueprint, item exposure limits, and related operational constraints.

This operational problem combines three correlated requirements. First, the parameters of a newly arrived item must be predicted from its text alone, because no response data are available at the moment the item is administered. Second, each prediction must be accompanied by an explicit estimate of its own uncertainty, so that the assembly step can separate items whose parameters are predicted reliably from those whose parameters are predicted poorly. Third, assembly must complete within a time limit on the order of one to a few seconds, which is the interval within which a student expects the next set of items to appear. These requirements constrain one another. In particular, the time limit rules out evaluating every possible item combination, and it rules out exact optimization methods that are accurate but slow. The uncertainty in the predicted parameters makes assembly a stochastic problem rather than a deterministic one.

The conventional procedure is incompatible with this loop. For example, traditional test assembly states item selection as a mixed-integer program (MIP), a form of constrained optimization that follows the blueprint and exposure rules, but it assumes the parameters are known and fixed. That is, each new item was administered to several hundred examinees in pretests before operational use. Therefore, what is needed is a single method that does two things together. First, it must predict the parameters of newly generated items from their text along with an explicit estimate of how uncertain each prediction is, and second, it must assemble each test within seconds while respecting operational constraints and carrying that uncertainty through to the final selection.

### 1.2. Reference Review

The dominant framework for automated test assembly is the MIP formulation introduced by van der Linden (2005) and refined through the shadow-test assembler tradition of van der Linden and Diao (2016). In this framework, form construction is treated as a yes-or-no selection decision for each item, made subject to linear constraints on content, test length, enemy items that must not appear together, and exposure, with an objective tied to the test information function (TIF) at one or more target ability levels. This framework underlies most modern computerized adaptive testing (CAT) programs, and it builds on the adaptive testing foundations of Owen (1975), the item response theory framework of Lord (1980) and Hambleton et al. (1991), and the exposure control procedures of Sympson and Hetter (1985) and Stocking and Lewis (1998). For calibrated items with stable parameter estimates, this framework delivers measurement precision and content fidelity that meet operational standards.

The framework rests on two assumptions that the personalized scenario violates. The first is that item parameters are known with negligible error. Veldkamp (2013) and Veldkamp et al. (2013) showed that calibration error from moderate-sized field tests already biases assembly decisions, and they introduced robust optimization extensions that draw on Ben-Tal et al. (2009) and Bertsimas et al. (2011). Robust optimization is a class of methods that account for uncertainty in the problem parameters by optimizing against a set of plausible parameter values rather than against a single point estimate. This plausible set is called an uncertainty set, and it is usually defined as a confidence region around the point estimate. A robust solution is one that performs acceptably for every parameter value in the uncertainty set, rather than one that performs best at the point estimate but degrades when that estimate is wrong. These extensions handle the modest uncertainty produced by field tests with several hundred examinees per item, but they were not designed for the much larger uncertainty that arises when parameters are predicted from item text with no response data. The second assumption is that computation time is not the limiting factor. Operational MIPsolvers handle assembly problems with several hundred items and standard constraints in seconds to minutes. Once the assembly must also satisfy chance constraints, which require a condition to hold with at least a specified probability, or optimize a worst-case objective over an uncertainty set, and once it must be repeated for every student in a personalized loop, the cumulative runtime becomes prohibitive. The limiting factor is therefore not the speed of a single solve at moderate bank sizes, which remains within seconds, but the combination of robust constraints that can render the program infeasible and the repetition of the solve for every student in the loop.

A parallel line of work has developed transformer-based prediction of item parameters from text. A transformer is the neural-network architecture that underlies modern LLMs, and here it is trained to map the text of an item to its parameters. Settles et al. (2020) demonstrated operational deployment of such prediction in the Duolingo English Test. Benedetto et al. (2021) reported that transformer predictions of difficulty for medical and educational multiple-choice items, although less accurate than calibrated estimates, were accurate enough to support operational decisions. Yaneva et al. (2020) applied this approach to high-stakes medical examinations. These studies show that transformer prediction of item difficulty is stable enough for operational use, but they stop at the prediction step and do not address how the subsequent assembly should use the predictions. In particular, they typically report the accuracy of point estimates without assessing whether the uncertainty attached to each prediction is reliable, and they do not connect prediction uncertainty to assembly decisions.

The literature reviewed above leaves a gap at the intersection of these three problems. MIP assembly treats parameters as known and pursues exact optimality without a time budget, so it does not address the case in which parameters carry prediction error. Robust optimization extensions accommodate uncertainty of the magnitude produced by field tests with several hundred responses per item and place robustness in the constraints, but feasibility shrinks as uncertainty grows, and the uncertainty that arises from text prediction is larger by an order of magnitude. Text-prediction studies report point accuracy without validating whether the attached predictive uncertainty is reliable, and they stop before the assembly step. So far, no study has joined validated predictive uncertainty with an assembly formulation that both uses that uncertainty and returns a form within a per-student time budget. In this regard, three capabilities need to work together to support real-time assembly when item parameters are predicted rather than calibrated. The first is text-based prediction of item parameters with trustworthy uncertainty estimates, meaning that a stated 90% predictive interval actually covers the true parameter value roughly 90% of the time in held-out item sets. Without this property, the assembly step cannot tell which predictions are reliable and which are not, and it must treat every item as equally uncertain. The second is a robust assembly formulation that carries each item’s uncertainty into the objective function and the constraints, producing forms that perform well on average while limiting the risk of poor realized measurement. The third is a solver that returns high-quality assembly solutions within a time budget measured in seconds. Ideally the solver exhibits anytime behavior, indicating it can be stopped at any point and will return the best solution found so far, so that the time budget is a tunable parameter rather than a hard threshold.

### 1.3. Proposed Method

In our proposed framework, the first capability is supplied by pairing a transformer regression model with established methods for estimating predictive uncertainty. A transformer is the neural-network architecture that underlies modern LLMs, and here it is trained to read the text of an item and output its predicted parameters. Two standard methods from the AI literature generate uncertainty estimates around these predictions, Monte Carlo dropout, following Gal and Ghahramani (2016), and deep ensembles, following Lakshminarayanan et al. (2017). Neither method has been systematically applied to psychometric calibration. Both work by producing many predictions for the same item through controlled randomness. For example, Monte Carlo dropout does so by randomly deactivating a fraction of the network’s internal connections each time a prediction is made and then repeating this process many times. Deep ensembles do so by training several independent copies of the network from different random starting values. In either case, the spread across the resulting predictions serves as an estimate of predictive uncertainty, analogous to the standard error that accompanies a calibrated item parameter estimate. The second capability is supplied by formulating the assembly problem with a mean–variance objective in the tradition of Veldkamp (2013) and Veldkamp et al. (2013), extended here to accommodate the larger uncertainty that text-only prediction produces. The third capability depends on the choice of solver, and it is here that the following proposed framework becomes important.

To explain this framework, it helps to start from the optimization problem it solves. The robust assembly problem, once uncertainty penalties and constraint penalties are written as squared terms, takes the form of a Quadratic Unconstrained Binary Optimization (QUBO) problem. In a QUBO problem, the decision is a vector of binary indicators, one per candidate item, where 1 means the item is selected and 0 means it is not. The objective function contains two kinds of terms: single-item terms that capture each item’s individual contribution, and pairwise terms that capture the interaction between every pair of items. Requirements such as test length, content coverage, enemy-item restrictions, and exposure limits are expressed as squared penalty terms in the objective rather than as separate constraints. A solution that violates a requirement incurs a large penalty, which drives the solver away from infeasible solutions.

The QUBO problem is mathematically equivalent to the Ising model from statistical physics, as established by Lucas (2014) and Glover et al. (2019). The Ising model has been used for analyzing item response data in psychometrics and educational measurement (Zhang & Chen, 2024). It describes a collection of binary variables, called spins, that each take the value −1 or +1. An energy function sums a linear field term for each spin and a pairwise interaction term for each pair of spins. Finding the spin configuration with the lowest energy is the same computational task as finding the item-selection vector with the lowest QUBO objective. The only difference is notation, that QUBO uses 0 and 1, and the Ising model uses −1 and +1, and a simple change of variables converts one into the other. Therefore, Ising-style solvers are algorithms designed to find low-energy configurations efficiently, and they include simulated annealing, parallel tempering, and modern hardware annealers.

Four properties make Ising-style solvers well suited to the personalized assembly scenario. First, these algorithms are anytime algorithms. This means that simulated annealing (Kirkpatrick et al., 1983), parallel tempering, and the digital annealer algorithms (Aramon et al., 2019) all produce a feasible solution within milliseconds and improve it continuously until stopped. Under a one-second time budget the solver returns whatever best solution it has found at the deadline of the budget, which is the behavior the personalized loop requires. MIP solvers, by contrast, often spend most of their time proving that an already-found solution is optimal. The chance-constrained and robust formulations of MIP, however, can become infeasible when the additional constraints shrink the feasible region under high uncertainty. Second, Ising-style solvers handle quadratic objectives and quadratic penalty terms natively, which matches the structure of the robust assembly problem. Translating the same problem into MIP form requires linearization, and each pairwise product of two item-selection indicators must be replaced by an auxiliary binary variable together with additional linear constraints so that a linear solver can process it. The number of auxiliary variables grows with the number of pairwise terms, which inflates the problem and slows the solve. Third, Ising-style solvers parallelize readily. Hundreds of independent annealing runs can execute simultaneously on a multicore processor, and the best feasible result is returned. This gives the assembly loop a direct way to trade hardware resources for solution quality within the time budget. Fourth, the QUBO formulation is compatible with dedicated Ising hardware such as quantum annealers and digital annealers (Mohseni et al., 2022), so a pipeline built against the QUBO interface could benefit from such hardware without changing the assembly objective. This compatibility is a direction for future work rather than a result of the present study, which uses a classical solver. These four properties distinguish Ising-style solvers from both general-purpose MIP solvers, which target exact optimality on smaller problems, and from greedy heuristics, which are fast but do not exploit the pairwise structure of the problem. For the robust real-time assembly problem addressed here, the Ising framework matches both the mathematical structure of the optimization and the operational demands of the deployment setting in operational assessments.

This study addresses three research questions. First, whether ensemble uncertainty yields predictive intervals whose empirical coverage matches their nominal level on an operational item bank? Second, whether carrying that uncertainty into the assembly objective reduces the discrepancy between reported and realized measurement precision, and how the reduction depends on prediction error magnitude and the share of predicted items in the bank? Third, whether an anytime solver returns feasible forms of acceptable quality within the per-student time budget imposed by the personalized assessment loop? The remainder of the paper is organized as follows. Section 2 develops the proposed method in full detail, covering the item response theory model, the transformer pipeline with uncertainty quantification, the QUBO formulation of robust assembly, and the Ising-style solving procedure. Detailed algorithmic information for each method can be found in the Supplementary Material. Section 3 presents the simulation study. Section 4 presents the empirical study on a state K–12 English Language Arts item bank. Section 5 discusses implications, limitations, and future directions. Section 6 concludes this study.

## 2. Methods

**Figure 1:**
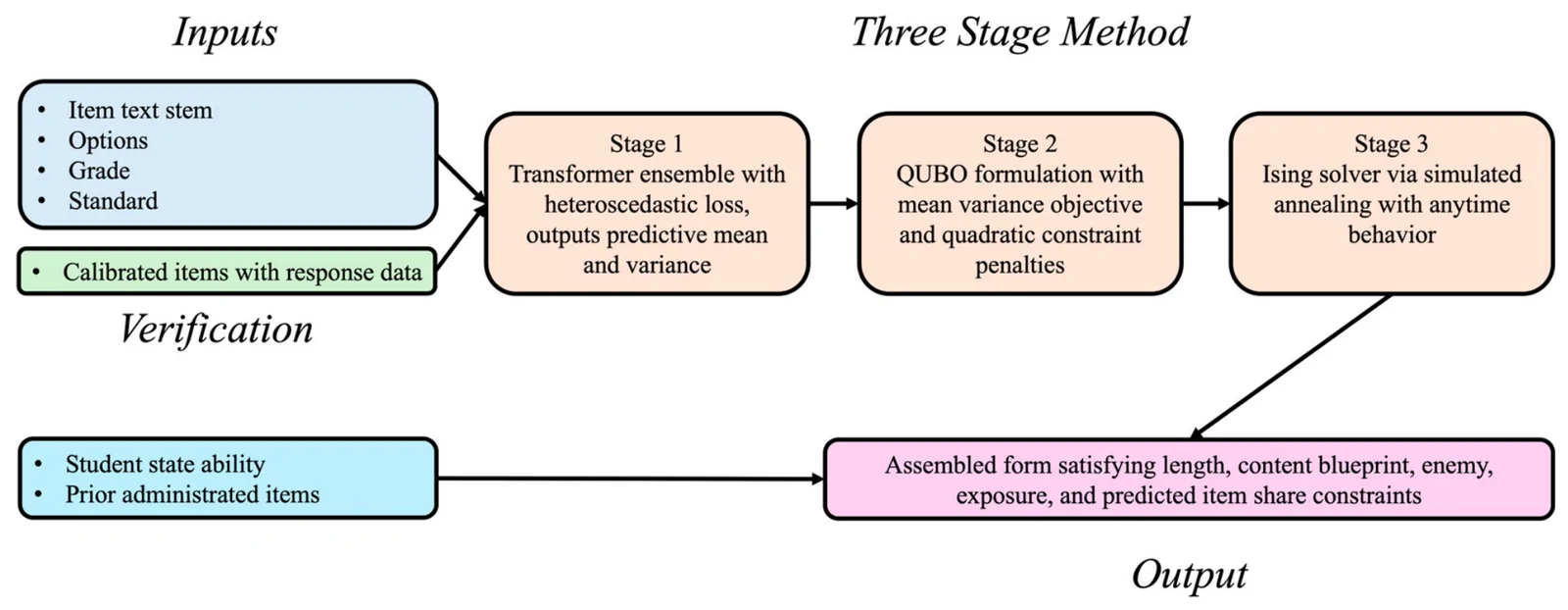
Three-stage frameworks for robust real-time test assembly under predicted parameter uncertainty.

We organize the method into three stages that together implement the personalized assembly loop. Stage 1 predicts item parameters from text and also produces associated uncertainty estimates. Stage 2 transforms the robust assembly problem into a QUBO problem, which was described in Section 1. Stage 3 solves the QUBO problem with an Ising-style algorithm within an operational time budget. Figure 1 summarizes the pipeline and Table 1 lists the notation used throughout in this manuscript.

### 2.1. Item Response Theory Model

The 2PL IRT model showing the probability that examinee j with ability $\theta_{j}$ responds correctly to item i with parameters $\left(a_{i},b_{i}\right)$ is

(1) $$P_{i}\left(\theta_{j}\right)=P\left(Y_{ij}=1|\theta_{j},a_{i},b_{i}\right)=\frac{exp\left[a_{i}\left(\theta_{j}-b_{i}\right)\right]}{1+exp\left[a_{i}\left(\theta_{j}-b_{i}\right)\right]}$$

The Fisher information contributed by item i at ability θ is given by

(2) $$I_{i}\left(\theta \right)=a_{i}^{2}\;P_{i}\left(\theta \right)\;\left[1-P_{i}\left(\theta \right)\right]$$

The test information function (TIF) for a form indexed by $x\in \{0,1{\}}^{N}$ can be calculated as

(3) $$TIF\left(\theta ;x\right)=\sum_{i=1}^{N}x_{i}\;I_{i}\left(\theta \right)$$

The asymptotic conditional standard error of measurement at θ is

(4) $$SEM\left(\theta ;x\right)=\frac{1}{\sqrt{TIF\left(\theta ;x\right)}}$$

For items in the calibrated set C, the parameters $\left(a_{i},b_{i}\right)$ are treated as known constants estimated from prior field-test data via marginal maximum likelihood (Bock & Aitkin, 1981). For items in the predicted set P, the transformer will produce a predictive distribution that we approximate as bivariate normal on the log-discrimination and difficulty scale, with mean $\left({\hat{\mu }}_{i}^{loga},{\hat{\mu }}_{i}^{b}\right)$ and covariance ${\hat{\Sigma }}_{i}$. The log-discrimination scale ensures positivity after exponentiation and is consistent with standard parameterizations used in marginal maximum likelihood estimation.

### 2.2. Stage 1: Transformer-Based Calibration with Uncertainty Quantification

We provide a brief conceptual overview of transformer models before describing the specific implementation. A transformer model is a deep neural network that processes a sequence of tokens, such as words or sub word fragments, by computing weighted associations among all positions in the sequence using a mechanism called self-attention. Self-attention can be understood as a learned, data-driven set of weights that quantify how strongly each word in an item stem informs the representation of every other word, similar in spirit to how factor loadings quantify how strongly latent traits inform observed indicators, except that here the weights are computed dynamically from the input rather than estimated as fixed parameters. Each token is first embedded as a high dimensional vector, then passed through a stack of attention and feed forward neural network layers that allow the model to integrate information from across the entire input. In this process, an embedding is a numerical vector that represents a word or token in a continuous high dimensional space, where words with similar meanings sit close to each other. The pretrained encoder used here, RoBERTa, was trained on a large corpus of text using a masked language modeling objective (Liu et al., 2021). The masked language modeling objective requires the model to fill in randomly hidden words from their surrounding context, which forces it to learn general-purpose representations of language without item-parameter labels. This pretraining produces an encoder whose representations can subsequently be reused for downstream tasks. For example, to predict item parameters, a short regression layer is added on top of the encoder. This regression layer takes the encoder’s numerical summary of the item text and maps it to predicted values of item discrimination and difficulty. The entire model is fine-tuned and trained jointly on items whose parameters have already been calibrated from response data, so that the model learns to associate patterns in item text with known parameter values.

#### 2.2.1. Architecture and Input Encoding

RoBERTa has stable performance on item text regression tasks (Benedetto et al., 2021). The input sequence for item i is constructed by concatenating the item stem, each answer option, the grade-level label, and the content-standard label, with separator tokens between segments. The maximum sequence length is 512 tokens, and longer inputs are truncated at the stem boundary while preserving the answer options.

The encoder processes this input and produces a single summary vector for the entire item. A two-layer regression component maps the summary vector to four outputs, the predicted mean of log-discrimination, the predicted mean of difficulty, and the predicted log-variance for each parameter. The log-variance output ensures that predicted variances are always positive. Dropout regularization is applied during training to prevent overfitting when the set of calibrated training items is small.

The pretrained RoBERTa-base encoder is fine-tuned end-to-end with the regression component. Early stopping on validation negative log-likelihood with a patience of three epochs prevents overfitting on small training sets. Five independently seeded models constitute the ensemble. The detailed training process and full hyperparameters are reported in Table S1 of Supplementary Section S1.

#### 2.2.2. Loss Function

Training minimizes the joint heteroscedastic Gaussian negative log likelihood, which simultaneously fits the predictive mean and the predictive variance. The term heteroscedastic means that the model is allowed to predict a different variance for each item, rather than assuming a single common variance across all items. The loss can be written as,

(5) $$L=\frac{1}{\left|D\right|}\sum_{i\in D}\left[\frac{{\left({\hat{\mu }}_{i}^{loga}-loga_{i}^{⋆}\right)}^{2}}{2{\hat{\sigma }}_{i}^{loga,2}}+\frac{1}{2}log{\hat{\sigma }}_{i}^{loga,2}+\frac{{\left({\hat{\mu }}_{i}^{b}-b_{i}^{⋆}\right)}^{2}}{2{\hat{\sigma }}_{i}^{b,2}}+\frac{1}{2}log{\hat{\sigma }}_{i}^{b,2}\right]$$

where D is the training set, $a_{i}^{⋆}$ and $b_{i}^{⋆}$ are ground-truth calibrated parameters for training items, and the two component losses are summed with equal weight. Each component has two parts. The first part is a weighted squared error in which the weight is the inverse of the predicted variance, so that items the model is uncertain about contribute less to the error term. The second part is a log-variance term that prevents the model from making its predicted variance artificially small, because doing so would drive this term toward negative infinity.

#### 2.2.3. Uncertainty Quantification

Two methods produce the predictive covariance matrix ${\hat{\Sigma }}_{i}$. The primary method is a deep ensemble of five independently initialized models trained on the same training set with different random seeds, following Lakshminarayanan et al. (2017). Letting ${\hat{\mu }}_{i,m}$ and ${\hat{\sigma }}_{i,m}^{2}$ denote the mean and variance from ensemble member m, the ensemble predictive mean for each parameter is given by,

(6) $${\bar{\mu }}_{i}=\frac{1}{M}\sum_{m=1}^{M}{\hat{\mu }}_{i,m}$$

and the ensemble predictive variance is,

(7) $${\bar{\sigma }}_{i}^{2}=\frac{1}{M}\sum_{m=1}^{M}\left[{\hat{\sigma }}_{i,m}^{2}+{\left({\hat{\mu }}_{i,m}-{\bar{\mu }}_{i}\right)}^{2}\right]$$

The first term in Equation (7) captures aleatoric uncertainty learned by each ensemble member, which reflects irreducible noise in the item parameter labels. The second term captures epistemic uncertainty across ensemble members, which reflects what the model does not know because of finite training data. They form the total predictive variance. The cross covariance between log-discrimination and difficulty is estimated by

(8) $$\hat{Cov}{\left(\log a,b\right)}_{i}=\frac{1}{M}\sum_{m=1}^{M}\left({\hat{\mu }}_{i,m}^{loga}-{\bar{\mu }}_{i}^{loga}\right)\left({\hat{\mu }}_{i,m}^{b}-{\bar{\mu }}_{i}^{b}\right)$$

which, together with the per-parameter variances, yields the full predictive covariance matrix ${\hat{\Sigma }}_{i}$ on the log-discrimination and difficulty scale.

The secondary method is Monte Carlo dropout (Gal & Ghahramani, 2016), in which dropout is retained at inference time and 50 stochastic forward passes are aggregated per item. The phrase inference time refers to the prediction step that follows training. A forward pass is one full evaluation of the network from input to output, and a stochastic forward pass is a forward pass in which a different random subset of the network connections is temporarily disabled. The 50 passes per item therefore generate 50 slightly different point predictions for the same item, and the mean and variance across these 50 values estimate the predictive mean and the predictive variance for that item. On the validation set, the deep ensemble produced coverage closer to the nominal level than Monte Carlo dropout, and the comparison is reported in Table S4 of the Supplementary Material. We therefore use the deep ensemble as the primary uncertainty method throughout.

#### 2.2.4. Recalibration

Empirical coverage of nominal $\left(1-\alpha \right)$ predictive intervals is evaluated on the validation set. If empirical coverage deviates from nominal by more than five percentage points at any of the reference levels α∈{0.05,0.10,0.20}, we fit an isotonic regression of empirical against nominal coverage on the validation set and apply it to test set predictions as a post hoc recalibration step. Isotonic regression is a monotone nonparametric regression that does not impose a functional form. The procedure follows Kuleshov et al. (2018) for isotonic recalibration of regression intervals, with the calibration assessment approach of Guo et al. (2017). The recalibrated predictive variance becomes the operational ${\hat{\Sigma }}_{i}$ used in the assembly stage. This recalibration is part of the method rather than a post hoc adjustment applied to one study.

### 2.3. Stage 2: QUBO Formulation of Robust Assembly

Stage 2 converts the robust assembly problem with predicted parameter uncertainty into a QUBO problem through two steps. The first step bridges Stages 1 and 2 by propagating item-parameter uncertainty into the TIF. The second step encodes the assembly constraints as quadratic penalty terms following the standard QUBO construction described by Lucas (2014) and the practical guidance of Glover et al. (2019).

#### 2.3.1. Propagating Parameter Uncertainty into the Information Function

The transformer ensemble in Stage 1 produces a predicted mean and a predicted variance for the log-discrimination and the difficulty of each item, but the assembly objective is written in terms of Fisher information at the target ability points, and Fisher information is a smooth but nonlinear function of these parameters. To bridge the two stages, we apply the delta method, which is a first order Taylor expansion of a function around the mean of its random inputs that approximates the variance of the function as the squared gradient at the mean times the variance of the inputs. In our setting, the function is the Fisher information at a fixed ability point, the inputs are the predicted log-discrimination and difficulty for an item, and the output is the predicted mean and predicted variance of that item’s Fisher information.

#### 2.3.2. Mean Variance Objective

For a set of T target ability points $\left\{\theta_{1},\ldots ,\theta_{T}\right\}$ with weights $\left\{w_{1},\ldots ,w_{T}\right\}$ satisfying $\sum_{t}w_{t}=1$, the mean-variance assembly objective can be written as,

(9) $$U\left(x\right)=\sum_{t=1}^{T}w_{t}\left[\sum_{i=1}^{N}\mu_{i}^{\left(t\right)}x_{i}-\lambda \sum_{i=1}^{N}\tau_{i}^{\left(t\right)}x_{i}\right]$$

Both terms are linear in the binary vector x because $x_{i}^{2}=x_{i}$ for binary variables. The risk aversion coefficient λ≥0 controls the trade off between maximizing expected information and minimizing variance from prediction uncertainty. When λ=0, the objective reduces to maximizing expected information, and the formulation behaves like the naive baseline. When λ is large, the objective heavily penalizes items with large predicted variance, and the formulation favors calibrated items even at the cost of expected information. The independence assumption $Cov\left[I_{i},I_{j}\right]\approx 0$ for i≠j is reasonable when transformer prediction errors are approximately conditionally independent given item text. This assumption can be evaluated by introducing correlated prediction errors at known levels for each empirical data.

#### 2.3.3. Constraint Penalties

Test assembly constraints are encoded as quadratic penalty terms that are added to the objective, following the standard QUBO recipe in Lucas (2014) and the practical guidance of Glover et al. (2019). Each penalty equals zero when the constraint is satisfied and strictly positive when it is violated, with the magnitude of the violation entering quadratically.

##### Test-Length Constraint

The exact-length constraint becomes

(10) $$H_{len}\left(x\right)=P_{1}{\left(\sum_{i=1}^{N}x_{i}-n\right)}^{2}$$

Expanding the square yields linear and pairwise terms in x, which is the standard QUBO form.

##### Content Blueprint Constraints

For each content standard k=1,…,K with item subset $S_{k}$ and target count $c_{k}$, the equality constraint becomes

(11) $$H_{cont}\left(x\right)=P_{2}\sum_{k=1}^{K}{\left(\sum_{i\in S_{k}}x_{i}-c_{k}\right)}^{2}$$

When blueprint constraints are specified as ranges $\left[{\underline{c}}_{k},{\bar{c}}_{k}\right]$ rather than exact counts, binary-encoded slack variables $s_{k}\in \{0,1,\ldots ,{\bar{c}}_{k}-{\underline{c}}_{k}\}$ are introduced and the penalty is written as

(12) $$H_{cont}\left(x\right)=P_{2}\sum_{k=1}^{K}{\left(\sum_{i\in S_{k}}x_{i}-{\underline{c}}_{k}-s_{k}\right)}^{2}$$

##### Enemy Item Constraints

For each enemy set $E_{l}$, in which at most one item may be administered together with any other item in the set, the constraint becomes

(13) $$H_{en}\left(x\right)=P_{3}\sum_{l}\sum_{i<j,\;i,j\in E_{l}}x_{i}x_{j}$$

which penalizes each pair of co-selected enemy items and yields a sparse pairwise interaction structure.

##### Predicted-Item Share Cap

The constraint that predicted items constitute at most a fraction f of the form becomes

(14) $$H_{pred}\left(x\right)=P_{4}{\left(\sum_{i\in P}x_{i}-f_{n}-s_{p}\right)}^{2}$$

with a binary expanded slack variable $s_{p}$.

##### Exposure Constraints

Per-item exposure caps over a window of W recent forms are handled by tracking cumulative usage $u_{i}$ and adding a per-item linear penalty $P_{5}$ that effectively makes heavily used items progressively more difficult to present in a selection candidate set. Let $u_{i}$ denote the cumulative count of administrations of item i across the past W forms. The exposure penalty is then defined as:

(15) $$H_{exp}(x)=P_{5}\sum_{i=1}^{N^{\prime }}u_{i}x_{i}$$

#### 2.3.4. Combined Objective

The full objective to minimize is obtained by negating the mean–variance (MV) objective so that lower values correspond to better measurement, then adding all constraint penalties, which coefficients $P_{1},\ldots ,P_{5}$ are calibrated as in Supplementary Section S3.

(16) $$H\left(x\right)=-U\left(x\right)+H_{len}\left(x\right)+H_{cont}\left(x\right)+H_{en}\left(x\right)+H_{pred}\left(x\right)+H_{exp}\left(x\right)$$

### 2.4. Stage 3: Ising Style Solving

The QUBO is solved by simulated annealing (Kirkpatrick et al., 1983), a general-purpose optimization algorithm well suited to binary selection problems. Specifically, the solver begins with a random set of selected items and iteratively improves the selection by swapping items in and out, guided by the combined objective in Equation (16). The algorithm is designed to avoid settling prematurely on a mediocre solution. Early in the search, it explores broadly across many possible item combinations, and it gradually narrows toward the best combination it has found. The solver runs many independent searches in parallel and returns the best feasible result across all of them, which provides a direct way to trade computation time for solution quality. Full algorithmic details are provided in Supplementary Section S4. The solver configuration and hyperparameters are reported in Table S2 of the Supplementary Material.

### 2.5. Real-Time Assembly

In operation, the three stages described above run as a closed loop for each student. When a student begins a session, the system retrieves the student’s current ability estimate, administration history, and target content standard. Any items that have been newly authored since the last session enter the bank with their transformer-predicted parameters and uncertainty estimates, and any items that have exceeded their exposure limit are retired. The system then assembles a short form tailored to the student’s current ability level by solving the QUBO, administers the selected items, updates the ability estimate from the responses, and repeats. Because the QUBO formulation treats calibrated and predicted items through the same interface, the assembly pipeline does not need to be restructured as newly generated items accumulate response data and transition from predicted to calibrated status. An item does not remain in predicted status indefinitely. Once it has accumulated 1000 responses, its parameters are re-estimated by marginal maximum likelihood, the predictive mean and variance from Stage 1 are replaced by the calibration estimate and its standard error, and the item moves to calibrated status. Because the objective in Equation (9) takes a mean and a variance for every item regardless of source, this substitution requires no change to the assembly formulation. The predicted share factor in the simulation should therefore be read as a snapshot of a bank at a given stage of maturity, where a share of 0.75 represents a bank in rapid expansion and a share of 0.25 represents one in which most items have accumulated response data. The framework assumes that items have passed content review before entering the bank, so that the assembly step is not responsible for quality control of item content. The full step-by-step procedure is given in Supplementary Section S5.

## 3. Simulation Study

### 3.1. Purpose and Design

To evaluate the proposed method performance, we use a fully crossed factorial design. Table 2 lists the factors and their levels. The factorial yields 5×3×3×2=90 combinations, with 100 replications for each combination.

For each combination, six solver methods are compared, as summarized in Table 3. Method M1 is a naive MIP assembly that treats predicted parameters as if they were exact. It serves as a baseline that ignores prediction uncertainty entirely. Method M2 is a chance-constrained MIP formulation at α= 0.10. Rather than requiring the test information function to meet its target exactly, this formulation requires only that the probability of falling below the target is at most α= 0.10, computed under the predicted parameter distribution. In other words, the assembled form is permitted to miss its information target, but only with at most a 10% chance. Method M3 is a robust MIP formulation based on the budget-of-uncertainty approach of Bertsimas and Sim (2003). This formulation assumes that at most a fixed number of items can simultaneously take their worst-case parameter values, and it assembles the form that performs best under that scenario. The approach remains tractable as an MIP because the worst case is defined over a simple geometric region rather than over the full predictive distribution. Method M4 is the proposed mean–variance (MV) QUBO solved by simulated annealing. Method M5 is a worst-case (WC) QUBO that replaces the MV objective with a one-sided lower confidence bound on each item’s information. It is conceptually similar to the Bertsimas and Sim approach but preserves the QUBO structure so that it can be solved by the same Ising-style solver as M4. Method M6 is a greedy heuristic that ranks items by their predicted mean Fisher information and selects items from the top of the list while respecting content and enemy-item constraints.

### 3.2. Data Generation

Each replication follows the procedure specified in Table 4. The simulation generates true item parameters by drawing discrimination and difficulty values from normal distributions, assigns each item to one of five content standards at fixed proportions, and creates an enemy-pair structure by clustering items on simulated stem similarity and sampling pairs within clusters. The bank is then partitioned into a calibrated set C and a predicted set P at the proportion specified by the design condition.

For each item in P, predicted parameters are generated by adding normal noise to the true values. The noise standard deviation increases across the low, medium, and high prediction-error conditions for both log-discrimination and difficulty, with exact values given in Table 4. In the well-calibrated uncertainty condition, the predicted variance equals the actual noise variance, so that the nominal coverage of predictive intervals matches the empirical coverage. A sensitivity branch introduces deliberate miscalibration by setting the predicted variance to twice or half the noise variance, representing overconfidence and underconfidence respectively.

Each method then assembles a form from the simulated bank, and outcomes are recorded. Target ability points, blueprint target counts, and the maximum share of predicted items per form are held constant across all conditions.

### 3.3. Metrics

For each method, we record the outcomes listed in Table 5. The primary outcome is the TIF gap, defined as the mean absolute difference between the true TIF and the claimed TIF across five ability points. For every method, the claimed TIF is the plug-in test information computed from the point parameters the method had access to, namely the calibrated parameters for calibrated items and the predicted means for predicted items. The variance estimates enter the assembly objective but not the claimed TIF, so the claimed TIF is defined identically across methods and the TIF gap is comparable across methods. Runtime is measured by wall-clock time. For proposed methods that produce anytime solutions, namely M4 and M5, we additionally record the best feasible solution at budgets of 0.1, 0.5, 1.0, 2.0, and 5.0 s. Methods that do not produce anytime solutions are run to natural completion or to a 60 s timeout. Additionally, a solution is counted as feasible only when every hard constraint is satisfied. A solution whose penalty terms are nonzero is counted as a violation regardless of the size of the penalty. This criterion is identical for the MIP and the QUBO methods, so feasibility is compared on the same basis across solvers.

The choice of the TIF gap as the primary outcome follows from Equation (4), which defines the conditional standard error of measurement as the reciprocal square root of the test information function. A discrepancy between the claimed and the true TIF is therefore a discrepancy in the standard error that the system reports for the resulting ability estimate. Ability RMSE and the TIF gap measure different properties. Ability RMSE measures how accurately the point estimate recovers true ability, whereas the TIF gap measures whether the precision the system claims for that estimate is correct. A method can perform well on ability RMSE and poorly on the TIF gap, because a form can produce accurate estimates while reporting a standard error that is too small. We treat the TIF gap as primary because every operational decision that consumes the reported standard error inherits its error, including adaptive stopping rules, classification decisions near cut scores, and judgments about whether a change in score between administrations is larger than measurement error. We report ability RMSE alongside it because the two do not always move together, and Section 3.4.5 examines the cases where they diverge.

### 3.4. Simulation Results

#### 3.4.1. TIF Gap by Method and Prediction Error Magnitude

**Figure 2:**
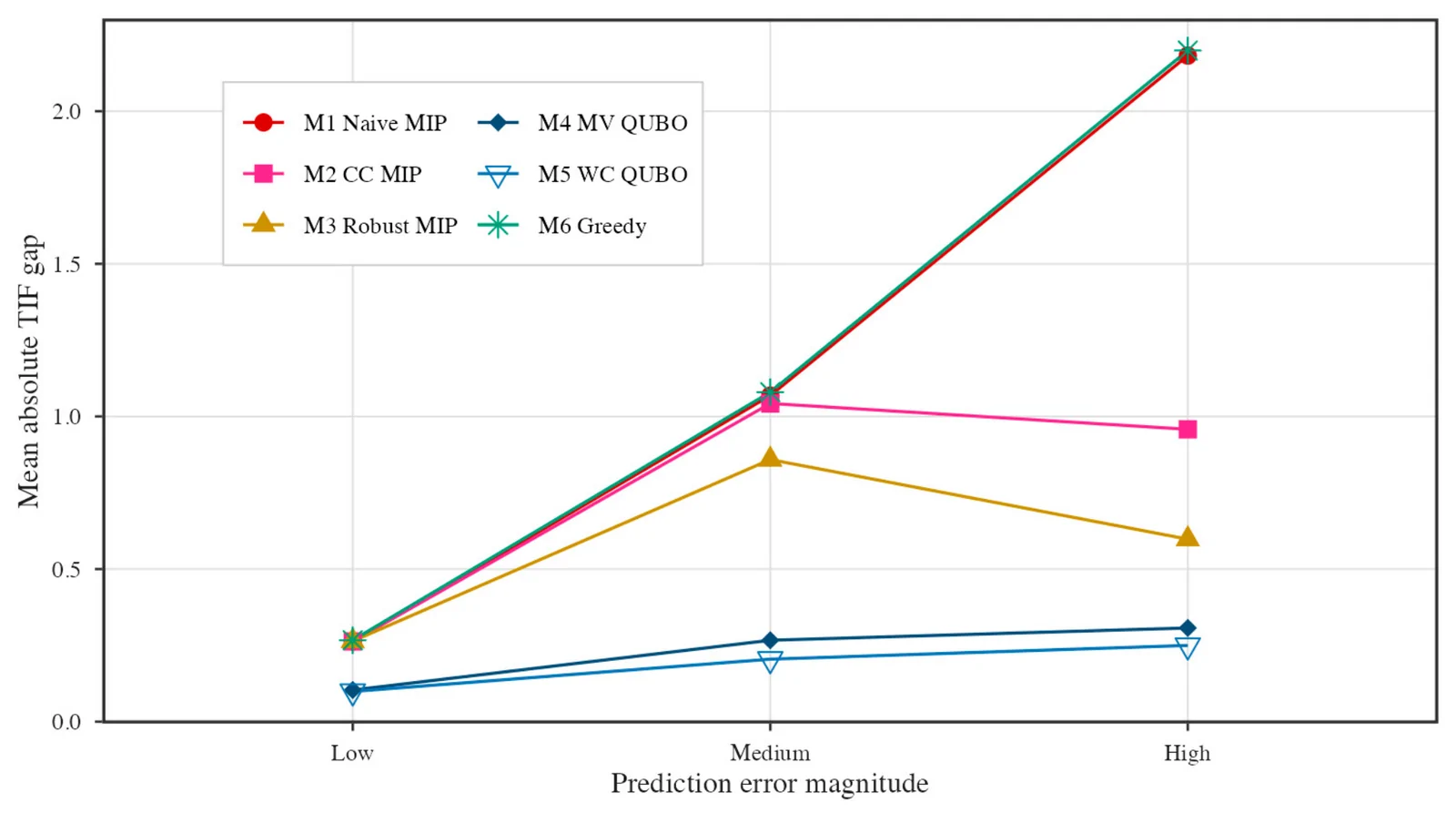
Method by prediction error magnitude interaction. The proposed methods M4 and M5 show flat growth in TIF gap as the prediction error magnitude grows from low to high. The naive methods M1 and M6 show steep growth.

Table 6 reports the mean absolute TIF gap by method and prediction-error magnitude, averaged over bank sizes and test lengths at predicted item shares of 0.25, 0.50, and 0.75. The proposed MV QUBO method (M4) achieves a mean gap of 0.226, and the WC QUBO method (M5) achieves the smallest mean gap at 0.185. The pattern is consistent across all three error magnitudes. Figure 2 depicts the method by prediction error magnitude interaction. It can be seen that the TIF gap for M4 and M5 remains nearly flat as prediction error increases from low to high, whereas the gap for the naive methods M1 and M6 grows steeply. Additionally, Supplementary Section S6 Table S3 reports the training and validation epoch at which early stopping halted training.

**Figure 3:**
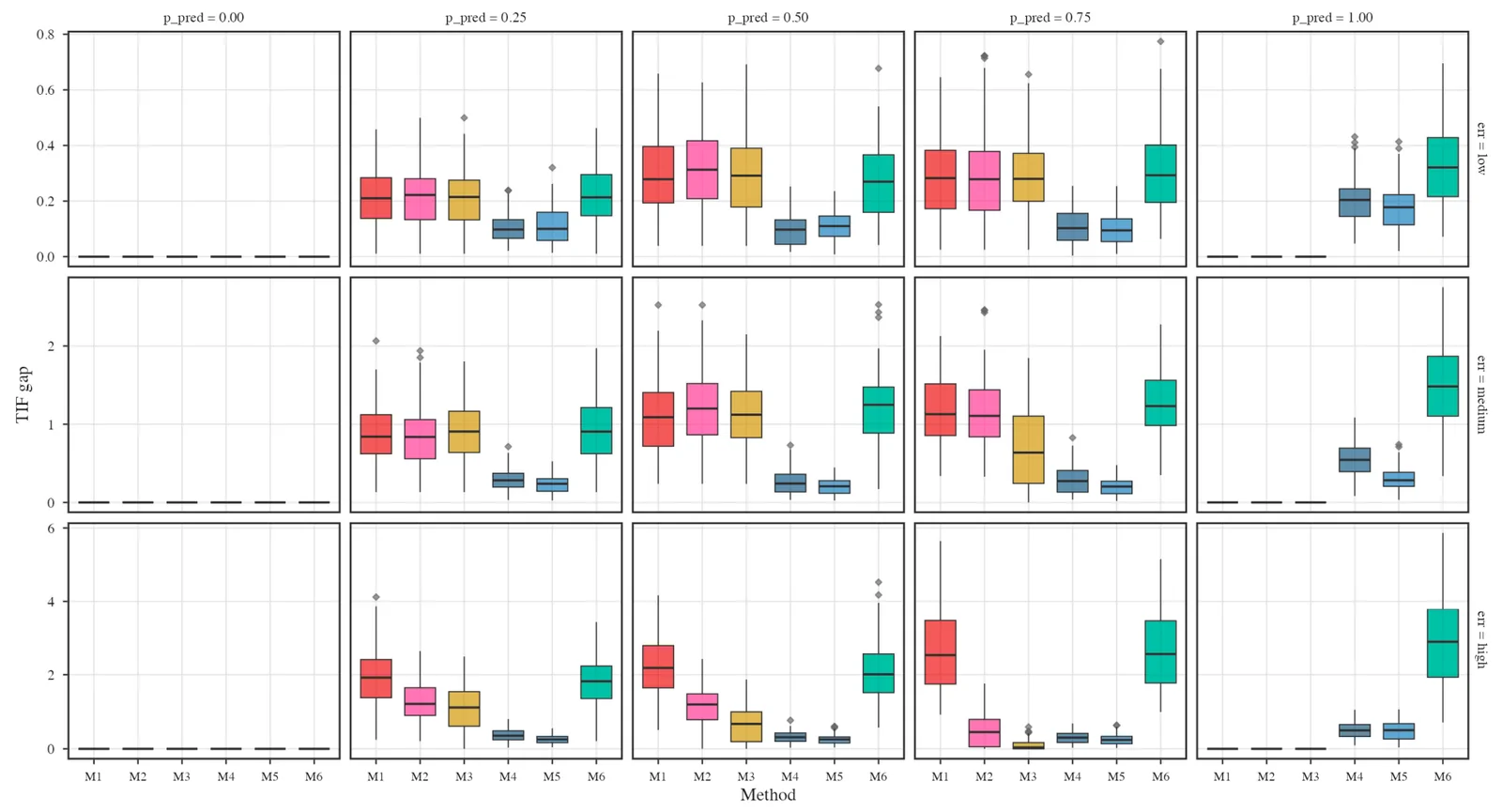
Distribution of TIF gap by method across the 15 factorial conditions defined by error magnitude and predicted item share. Boxes show the interquartile range, the central line marks the median, and whiskers extend to 1.5 times the interquartile range. Shape diamonds are outlier values. The proposed methods M4 and M5 hold tight distributions even at the highest error and highest predicted item share.

Figure 3 shows the distribution of TIF gap for each method across all factorial conditions. The advantage of M4 and M5 over the baseline methods widens as both prediction error and predicted item share increase, with the largest differences appearing under high error at predicted item shares of 0.25 and 0.50. The robust MIP method M3 narrows the gap relative to M1 at high prediction error, but its results become more variable and it frequently fails to produce a feasible form at a predicted item share of 0.75. When the predicted item share is zero, all methods produce a TIF gap of zero because no predicted items enter the form. When the predicted item share is 1.00, every method shows a constraint violation rate of 1.00, because the maximum predicted-item share constraint is set at 0.30 and cannot be satisfied when the entire bank consists of predicted items. This column falls outside the operational regime.

The pattern in Table 6 and Figure 3 has a straightforward explanation. When predictions are accurate, the variance penalty in the MV objective is small, so uncertainty-aware and naive methods select similar forms. When predictions are noisy, however, a naive method still selects items with the highest predicted information, ignoring the fact that those predictions are also the most uncertain. However, the uncertainty-aware methods, including M4, accept somewhat lower expected information in exchange for lower variance, so the true TIF of the assembled form tracks the claimed TIF more closely. When prediction error is zero or the predicted item share is zero, all methods produce equivalent forms.

#### 3.4.2. Solver Scaling and Anytime Behavior

**Figure 4:**
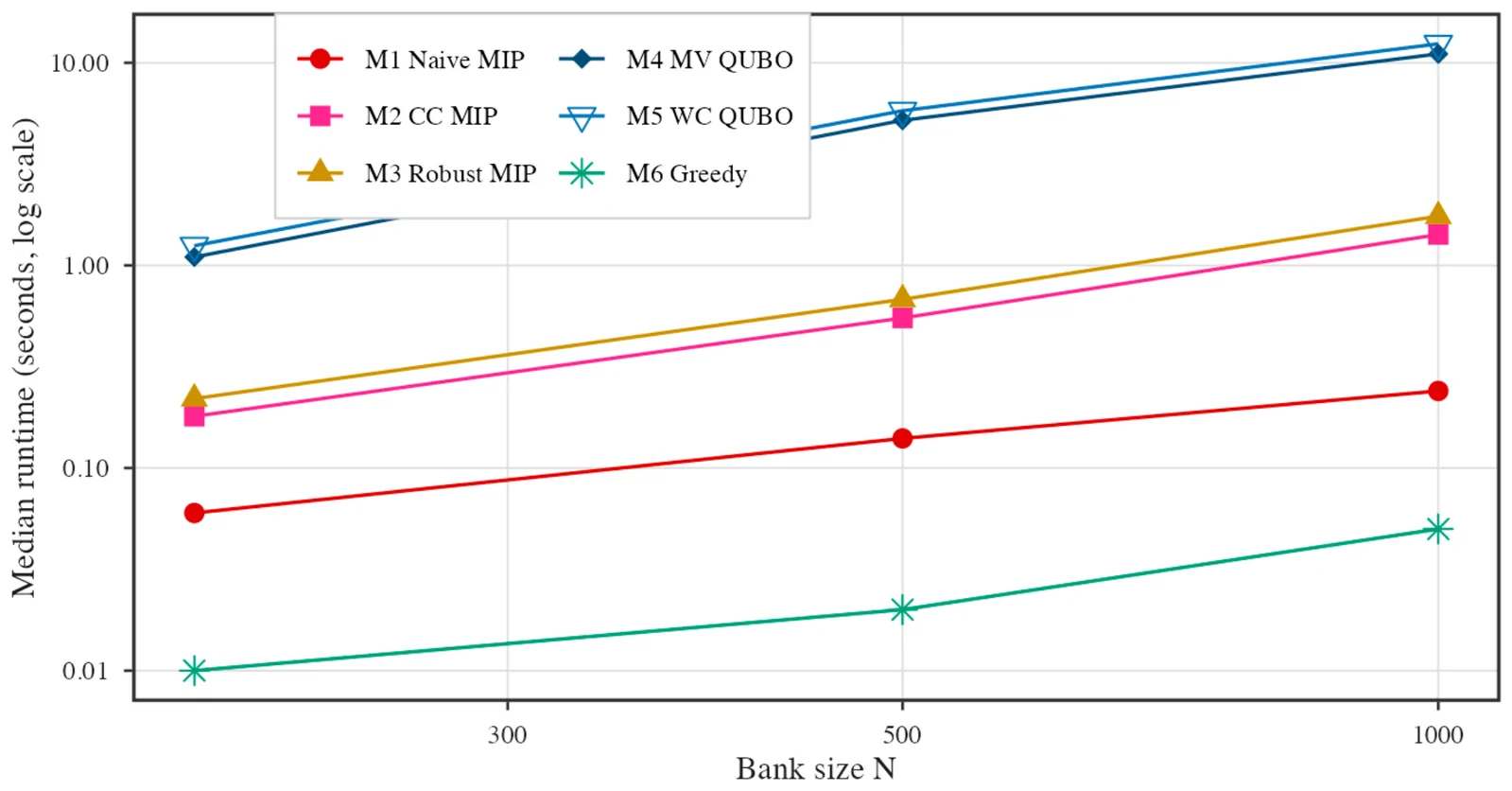
Runtime versus bank size on a logarithmic scale. The QUBO methods scale linearly with bank size.

Runtime grows roughly linearly with bank size for the naive and robust MIP methods M1 through M3 and more steeply for the QUBO methods M4 and M5. At N= 1000, M4 requires a mean of 11.06 s to run to completion, compared with 0.24 s for M1, 1.42 s for M2, and 1.75 s for M3, as shown in Table 7 and Figure 4. At the bank sizes studied, the MIP methods therefore complete within or near the one-second budget, and the proposed method is slower to completion. In practice, the argument for an anytime solver does not rest on faster completion. It rests on the behavior under a fixed time budget on highly constrained problems, where the personalized loop takes the best solution found so far rather than waiting for completion, and where a method that returns no feasible incumbent in time is more costly than one that returns a feasible form of moderate quality.

**Figure 5:**
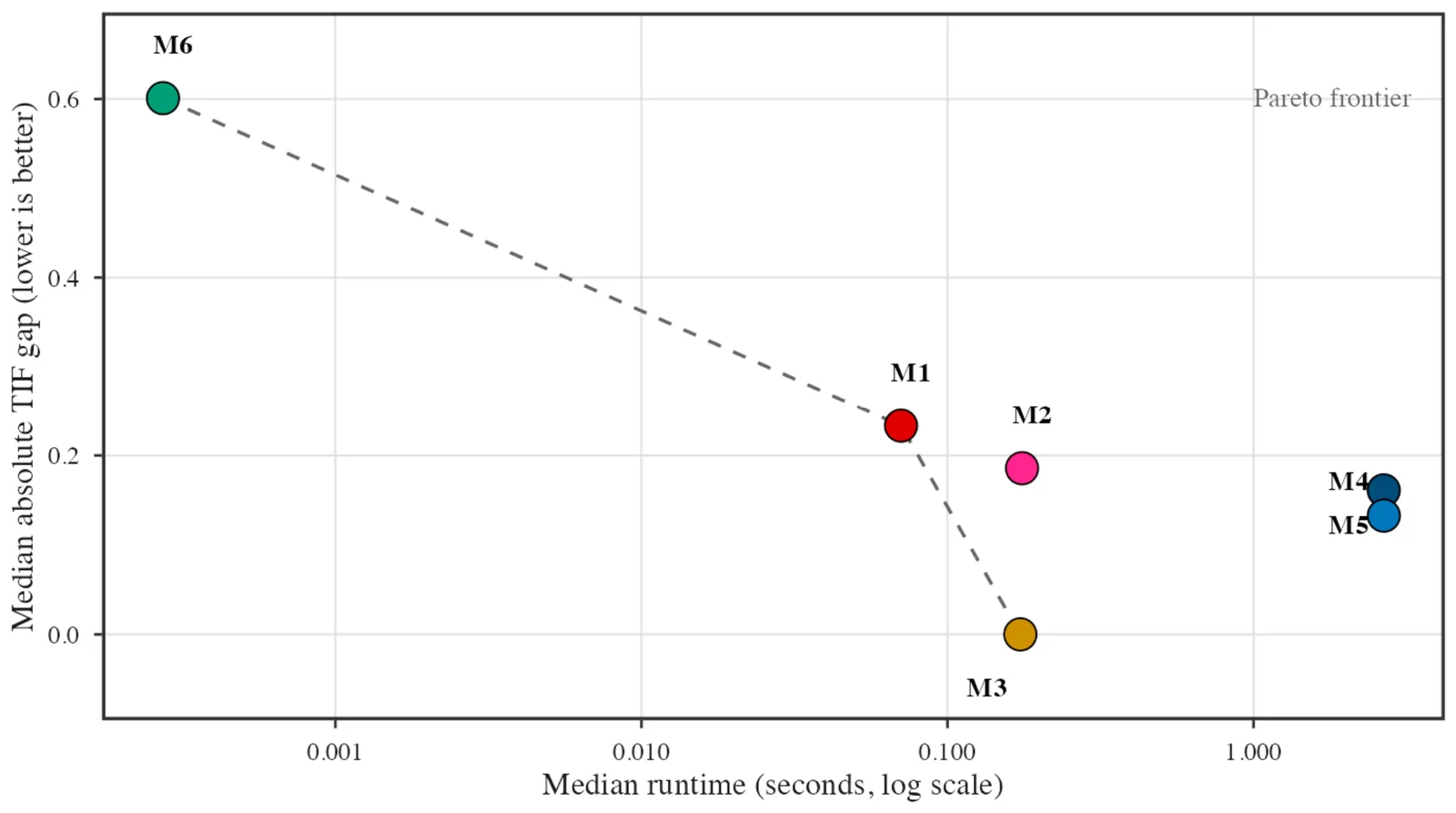
Accuracy versus runtime at bank size 1000, with method labels and the empirical Pareto frontier shown as a dashed line.

Figure 5 plots median TIF gap against median runtime at bank size 1000 and marks the empirical Pareto frontier. The Pareto frontier identifies the methods for which no other method is simultaneously more accurate and faster, and a method off the frontier is strictly outperformed by at least one method on it. For example, M4 and M5 sit in the high-accuracy region of the frontier at moderate runtime. M3 also reaches low TIF gap at sub-second runtime. M1 and M6 are fast but less accurate. Between the two proposed methods, although both are reasonable choices for operational use, M5 is slightly more accurate than M4 at comparable runtime, making the worst-case formulation preferable when a bound on the realized standard error of measurement is needed.

All simulation and empirical analyses were run on SageMaker instance ml.m5.8xlarge with 32 vCPUs and 128 GB. The MIP was solved with IBM ILOG CPLEX Version 22.1+, and the QUBO was solved with SimulatedAnnealingSampler from dwave-neal version 0.6.0.

The comparison in Table 7 runs each solver to completion, but completion does not mean the same thing for the two solver families. An MIP completes when optimality has been proven, whereas simulated annealing completes when its configured schedule of reads and sweeps is exhausted. The 11.06 s recorded for M4 at a bank size of 1000 therefore reflects the schedule of 200 reads at 10N sweeps that we set for the scaling analysis rather than a requirement of the problem, and the operational configuration reported in Table S2 of the Supplementary Material uses a one-second budget instead. What matters for the personalized loop is not how long the full schedule takes but how good the solution is when the time budget arrives, so Table 8 reports the best feasible solution available at each budget at the largest bank size studied, together with the converged solution as a reference.

At the one-second budget the proposed method reaches 94.0 percent of its converged information and a mean TIF gap of 0.247, compared with 0.224 at convergence, and a feasible solution satisfying every hard constraint was available in 99.4 percent of instances. The remaining improvement between one second and convergence is therefore modest: 6.0 percent in information and 0.023 in the TIF gap, which indicates that the one-second solution is operationally adequate for a real-time loop. The same pattern holds for M5. Because the solver is warm-started from the previous form in deployment, as described in Supplementary Section S4 and Algorithm S2, and because these simulation runs use cold starts, the values in Table 8 understate the quality available in an operational loop where the bank changes incrementally between calls.

**Figure 6:**
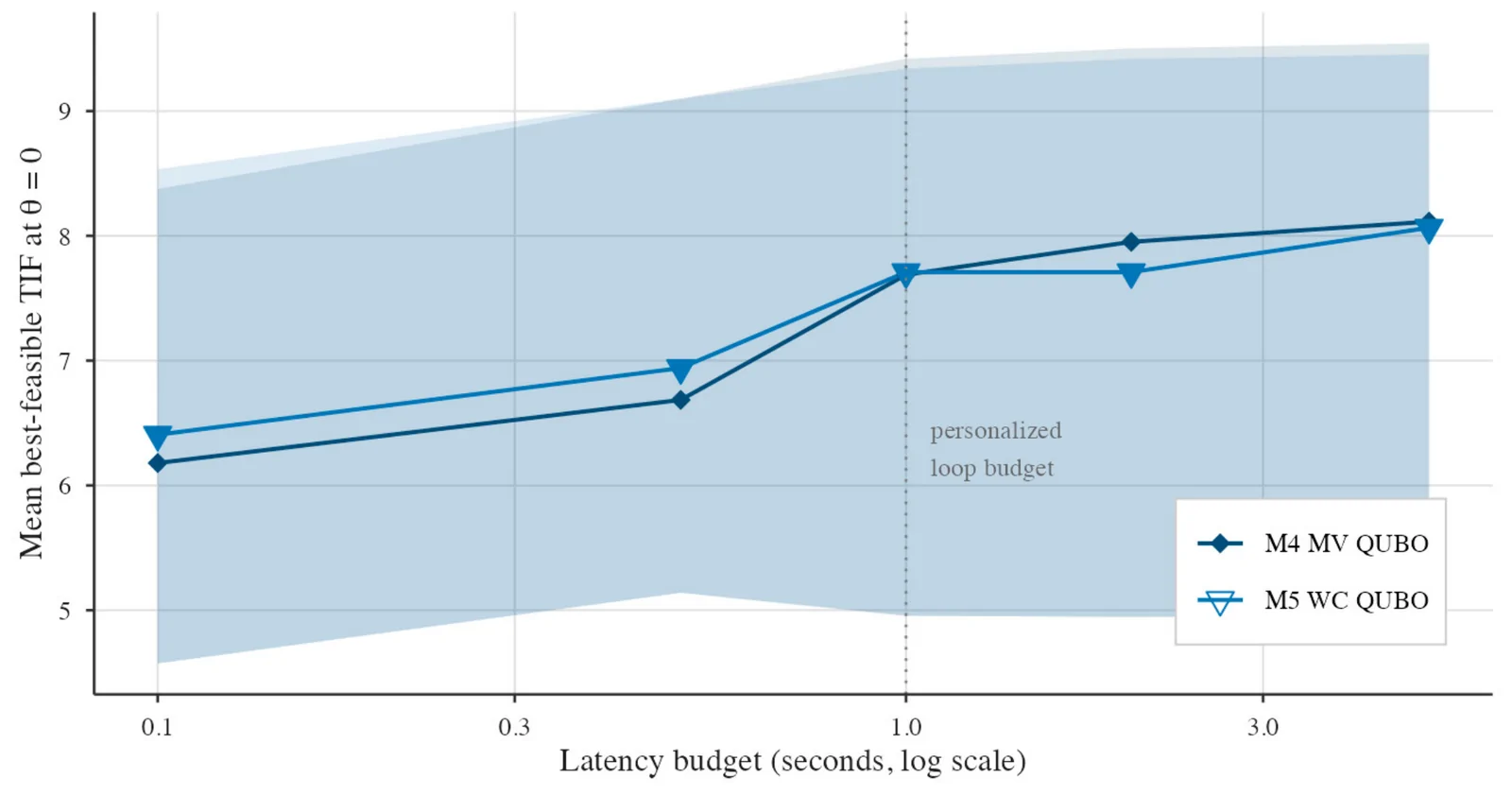
Anytime solution quality at increasing time budgets. The vertical dotted line marks the one-second operational budget. Shaded bands mark the interquartile range across conditions.

Figure 6 presents the mean test information at the five target ability points for the best feasible solution found at each time budget, computed from the parameters available to the solver. The shaded bands mark the interquartile range across conditions. Higher values indicate more information, and this metric is the total information level rather than the TIF gap reported elsewhere. The values for M5 are unchanged between the 1.0 and 2.0 s budgets because, in many replications, the best feasible solution found at 1.0 s is not improved before 2.0 s, and they rise again at 5.0 s as additional restarts find better solutions. Small non-monotonicities of this kind are expected when a metric is averaged across conditions over a stochastic restart procedure. It can be seen that the range across conditions are close to the mean value. This pattern indicates that the simulated annealing solver has largely converged within the operational time budget, confirming that the QUBO methods can deliver near-final measurement quality under real-time constraints. Specific values are shown in Table S5 in Supplementary Material.

#### 3.4.3. End-to-End Latency

The runtime results reported so far cover the assembly solve alone, but the operational claim concerns the full loop, so we profiled every component. Transformer prediction does not run inside the loop. An item is encoded once when it enters the bank, the five-member ensemble produces its predictive mean and covariance, and both are cached, so the per-call cost is limited to retrieving the cached values, evaluating item information and its variance at the three target ability points, building the QUBO matrix, solving it, and validating the result. Table 9 reports each component on SageMaker instance ml.m5.8xlarge with 32 vCPUs and 128 GB. The total per-call latency is 991.1 ms at a bank size of 200 and 999.7 ms at a bank size of 1000, against a budget of one second. Amortized transformer prediction costs 58.4 ms per item at ingestion, which is incurred once per item rather than once per student. We also note that the per-call candidate set in a personalized loop is not the full bank, because the system filters to the student’s grade, target content standard, and exposure-eligible items before assembling. The per-grade banks in our empirical study run from 190 to 270 items, so a bank size of 1000 represents a stress condition rather than a typical per-call size.

Table 10 shows what the proposed solver achieves at a fixed time budget, but it does not show how the alternatives behave under the same constraint, so we repeated the primary comparison with a hard one-second wall-clock cap applied to every method. Any method that had not returned a solution when the time budget arrived was recorded as returning nothing for that instance. Under this constraint, M4, M5, and M6 return a solution in all cases, M1 in 98.9%, M2 in 87.1%, and M3 in 64.7%. The ordering on TIF gap is M5, M4, M3, M2, M1, then M6 from smallest to largest. This makes the time budget a design factor rather than an assumption, and it isolates the practical question of what a personalized system actually receives when it calls each method.

#### 3.4.4. Constraint Satisfaction

Constraint satisfaction is an operational requirement that is often understated in test assembly comparisons. In a personalized testing loop, a method that fails to return a form is more costly than one that returns a form with moderate TIF gap, because the system has no fallback when assembly fails. Constraint satisfaction is therefore a critical practical criterion. For each instance, we recorded whether any hard constraint was violated, counting outright solver infeasibility as a violation.

**Figure 7:**
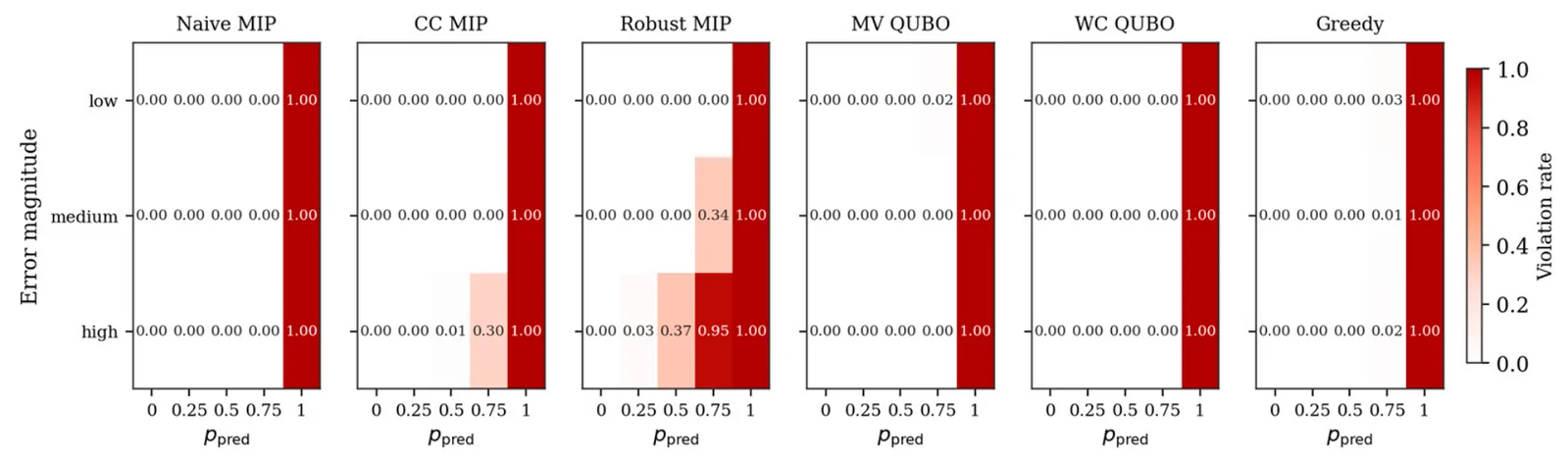
Constraint violation rate by method across factorial conditions. Each panel is one method. Within a panel, rows index error magnitude from low to high and columns index predicted item share from 0 to 1. Darker shading marks a higher rate of forms that violated one or more hard constraints.

Figure 7 displays the violation rate per factorial condition as a heatmap, with rows for error magnitude and columns for predicted item share. Two patterns stand out from the results. First, when the predicted item share is 1.00, every method shows a violation rate of 1.00. It is expected that the maximum predicted-item share constraint is set at 0.30, which cannot be satisfied when the entire bank consists of predicted items. This column is included for completeness but falls outside the operational regime. Second, within the operational regime where the predicted item share is below 1.00, M4 and M5 maintain violation rates at or near zero across conditions. The robust MIP method M3, by contrast, shows a mean violation rate of 0.141, rising to 0.95 at high error with a predicted item share of 0.75. Although M3 reaches low TIF gap at sub-second runtime from earlier results, Figure 7 indicated it fails to produce feasible forms in a substantial share of conditions. The naive MIP method M1 and the greedy heuristic M6 remain near zero, and the chance-constrained method M2 falls between these at 0.026.

The feasibility difference between the robust MIP method M3 and the QUBO methods follows from where each method places the robustness, not from the solver. M3 enforces robustness as additional hard constraints, which shrink the feasible region as uncertainty grows until, at high error and a high predicted-item share, no form satisfies all constraints simultaneously and the program is infeasible. The MV methods place robustness in the objective and leave the original feasible region unchanged, so they return a feasible form whenever one exists. This distinction is independent of the solver, because an integer linear program with the same objective and the same hard constraints retains the same feasible region as M4. When no feasible form exists, no method can construct one, so the comparison should be read as constraint-based robustness against objective-based robustness rather than as MIP against QUBO.

#### 3.4.5. Examinee Ability Recovery

The TIF gap measures how closely the assembled form’s true measurement precision matches its claimed precision, but the quantity of primary interest is the accuracy of the resulting ability estimates. We therefore simulated 100 examinees per assembled form, drawn from a standard normal ability distribution, and computed the RMSE of the maximum likelihood ability estimate relative to the true ability.

**Figure 8:**
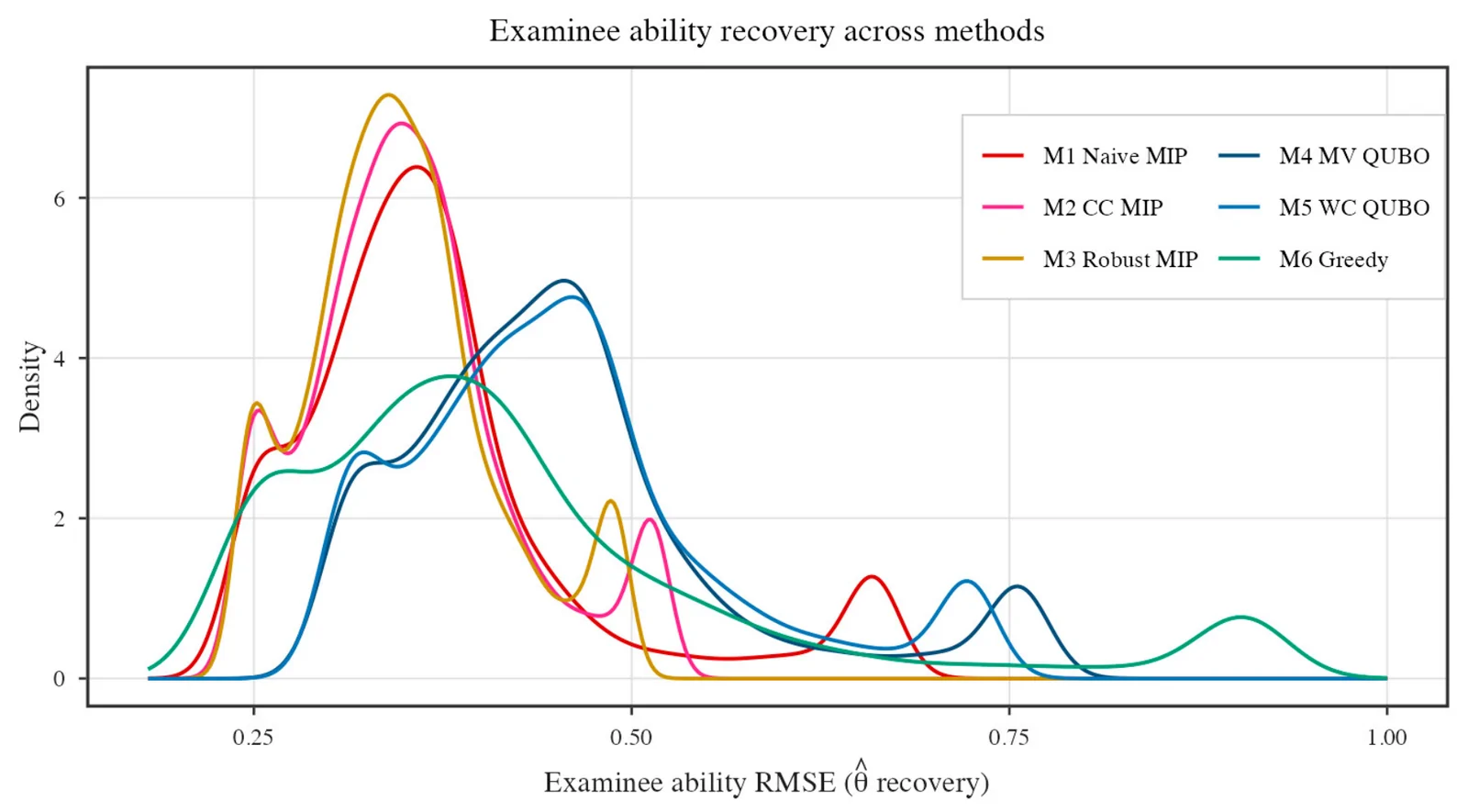
Density of examinee ability RMSE across feasible conditions of the factorial. Lower is better. The MIP methods M1 through M3 sit at lower RMSE within the conditions where they remain feasible. The QUBO methods M4 and M5 produce feasible forms in these conditions and sit at modestly higher RMSE.

Figure 8 shows the density of ability RMSE across all feasible cells of the factorial. The results reveal a trade-off that the TIF gap alone does not capture. In conditions where M1, M2, and M3 remain feasible, ability RMSE is concentrated near 0.34. The QUBO methods M4 and M5 produce ability RMSE near 0.44 across conditions. An error standard deviation of 0.34 corresponds to a reliability of approximately 0.90 and one of 0.44 to approximately 0.84, so the proposed method gives up roughly six points of reliability in the conditions where the mixed-integer methods remain feasible. This is not negligible, and we report it as a genuine cost rather than a rounding difference. What is gained in exchange is that the reported standard error is close to the realized one, as Table 11 shows. The proposed methods therefore accept a modest reduction in ability recovery in exchange for a substantially lower TIF gap and guaranteed feasibility across the full operational region. The greedy heuristic M6 falls between, performing well in easy conditions but degrading sharply in difficult ones. Importantly, the QUBO methods produce feasible forms, whereas the MIP methods fail in many conditions depending on the method.

Table 11 places the two quantities side by side. The naive method achieves a lower realized ability RMSE but reports a standard error of 0.270, understating its own error by 0.070. The proposed method achieves a higher realized RMSE but reports 0.421, which departs from the realized value by 0.019. This is what the TIF gap results describe, expressed on the scale of the standard error rather than on the scale of information. A system using the naive method would report a precision it does not deliver, whereas a system using the proposed method reports a precision close to what it delivers, even though that precision is lower.

Ability is estimated by maximum likelihood in the simulation and by expected a posteriori estimation in the empirical study. Because expected a posteriori estimation shrinks estimates toward the prior mean, it lowers RMSE near the center of the distribution and introduces bias at the extremes, so the tail results in Table 12 and those reported in Section 4.4 should be compared with that difference in mind rather than read as replications of one another.

The pooled figures conceal a pattern across the ability range. Table 12 shows that the naive method’s advantage in RMSE is concentrated at the center and near-center levels, while at θ of −2 and 2 the difference is small and reverses, with M4 lower by about 0.03. Bias follows the expected symmetric shrinkage toward zero for all methods, with smaller absolute bias under M4 and M5. This matters for the trade-off, because instructional placement decisions are made at the ends of the distribution more often than at the center, and the empirical study in Section 4.4 finds the proposed method ahead at the extremes.

Therefore, Figure 3, Figure 7 and Figure 8 show that the proposed methods guarantee feasibility and achieve a substantially lower TIF gap, at the cost of modestly higher ability RMSE in the easier conditions where MIP methods remain feasible. However, for a personalized testing program that must return a form on every call within a fixed time limit, feasibility is the decisive criterion. For a batch assembly program that can retry with a different solver when a condition fails, the MIP methods remain useful where they succeed. The contribution of the proposed approach is therefore best characterized as a joint advantage in feasibility and TIF accuracy rather than as uniform dominance on every metric.

#### 3.4.6. Sensitivity to Uncertainty Calibration

The results reported so far were produced under the well-calibrated uncertainty condition, in which the predicted variance assigned to each item equals the variance of the noise actually added to its parameters. Because the proposed objective penalizes items in proportion to their predicted variance, the quality of that variance, and not only its average size, can affect which items are selected. To isolate this effect, we held the predicted means fixed and rescaled only the predicted variance, setting it to half the true noise variance to represent overconfidence and to twice the true noise variance to represent underconfidence. All other factors were held at the levels used in the main analysis.

Table 13 summarizes the effect on the proposed MV QUBO method M4, with the naive MIP method M1 included as a reference because it ignores the predicted variance entirely. Coverage of the predictive intervals responds to the manipulation as expected. At the 90% nominal level, empirical coverage is 0.90 in the well-calibrated condition by construction, falls to 0.74 under overconfidence because the intervals are too narrow, and rises to 0.97 under underconfidence because the intervals are too wide. The downstream effect on measurement is asymmetric. Under overconfidence the mean absolute TIF gap of M4 increases from 0.226 to 0.402, whereas under underconfidence it decreases slightly to 0.198. The naive method is unchanged at 1.171 across all three conditions, which confirms that the manipulation acts only through the variance channel and not through the predicted means.

Table 14 shows that the degradation under overconfidence is concentrated at higher prediction error. The TIF gap of M4 under overconfidence rises from 0.142 at low error to 0.643 at high error, whereas under underconfidence it remains close to the well-calibrated values across all three error magnitudes. The examinee-level results follow the same ordering. Ability RMSE for M4 is 0.44 in the well-calibrated condition, 0.50 under overconfidence, and 0.47 under underconfidence, so both forms of miscalibration reduce ability recovery relative to correct calibration, but overconfidence does so more severely.

#### 3.4.7. Effect of Correlated Prediction Errors

The delta-method propagation in Equation (8) assumes that prediction errors are uncorrelated across items, which is unlikely to hold when items share a reading passage, an item family, or a common writer, and when all predictions come from a single encoder whose representations are shared. To test how far the framework degrades when that assumption fails, we regenerated the simulation with prediction errors drawn from a multivariate normal in which off-diagonal correlations were set to 0.0, 0.2, 0.4, and 0.6. A single correlation across all item pairs represents a stronger form of dependence than the passage-sharing or content-sharing case, because it applies to every pair rather than only to items within a shared group. Table 15 shows that as correlation rises from zero to 0.6, the TIF gap for the proposed method moves from 0.213 to 0.958 and interval coverage moves from 0.900 to 0.668. Because the variance of a sum of correlated terms exceeds the sum of the variances, the framework understates the true variance of the test information function by a factor that grows with the correlation, so the ordering of methods is preserved through 0.6 and the advantage does not disappear within the examined range.

#### 3.4.8. Sensitivity to Calibrated Item Variance

Our original formulation treated calibrated item parameters as known constants and assigned them zero information variance, which understates the total uncertainty of any form containing them and, more seriously, tells the solver that calibrated items carry no risk at all. Because the mean-variance objective penalizes items in proportion to their variance, this creates an incentive to load the form with calibrated items that is not justified by the data, and since the TIF gap is driven largely by how many predicted items reach the form, it inflates the apparent advantage of the proposed method. We therefore re-ran the analysis with each calibrated item assigned the information variance implied by its marginal maximum likelihood standard errors, propagated through the same delta method used for predicted items. Table 16 shows that under this correction the TIF gap for the proposed method moves from 0.158 to 0.213 while the naive baseline remains at 1.495, and the number of predicted items reaching the form changes from 3.2 to 4.6. The ordering of methods is preserved, which indicates that accounting for calibration error attenuates, but does not eliminate, the TIF-gap advantage of uncertainty-aware assembly.

#### 3.4.9. Shortfall Probability

The TIF gap reported above measures the average size of the discrepancy between claimed and realized information, but it does not distinguish a form that delivers more information than promised from one that delivers less. Only the second case threatens score interpretation, because a form whose realized information falls below the claimed target reports a standard error smaller than the one that actually applies. Table 17 therefore reports the proportion of forms in which this shortfall occurred. At a predicted share of 0.50 the naive baseline produced a shortfall in 69.0 percent of forms while the proposed method produced one in 22.0 percent, and the difference widens as the predicted share rises to 0.75. This is the quantity that robust assembly formulations are designed to control, so reporting it places our results in terms that readers of that literature can interpret directly.

### 3.5. Summary of Simulation Findings

In this simulation study, naive assembly that ignores prediction uncertainty produces substantially worse realized measurement quality than uncertainty-aware assembly, and the gap widens as prediction error increases. The pattern from sensitivity analysis has a direct explanation in terms of the objective. When the predicted variance is too small, the variance penalty is understated, so the solver under-penalizes items whose parameters are in fact uncertain and selects them as though they were reliable. Additionally, the WC QUBO method (M5) achieves the lowest TIF gap among all feasible methods across the operational conditions, with the MV QUBO method (M4) close behind. We now see that the QUBO methods produce feasible forms, whereas the robust MIP method and the naive MIP method fail in many conditions across the full design. Finally, the anytime behavior of the Ising-style solver allows the proposed methods to reach near-final quality within a one-second time budget, which is the constraint imposed by the personalized loop and the point at which MIP methods with chance constraints become too slow. These findings support the central claim of this study that the integration of transformer-based prediction with Ising-style robust assembly meets the operational requirements of real-time test assembly under continuous item bank growth.

## 4. Empirical Study

### 4.1. Item Bank

This empirical study uses a state-level English Language Arts item bank containing dichotomously scored multiple-choice items administered to students in Grades 3 through 12 in the United States. All items were calibrated under a 2PL model prior to this study, and the resulting parameters serve as gold-standard values for evaluation, although they were by marginal maximum likelihood with standard errors. Treating them as exact is a simplification, and the true TIF computed from them is therefore an estimate rather than an exact reference. The calibration standard errors are small relative to the prediction uncertainty studied here, because each item was calibrated on several hundred responses, but they are not zero.

Items were screened for inclusion on three criteria. The first criterion is that the estimated discrimination is greater than 0.3 and the estimated difficulty has absolute value less than 4. The second criterion is that both infit and outfit mean square statistics fall within 0.7 and 1.3 as a unidimensionality check. The third criterion is no substantial differential item functioning by gender, defined as a Mantel–Haenszel difficulty effect size below 0.5. For each item, the stem text, answer options, grade level, and content standard are used as input features for parameter prediction.

### 4.2. Model Pipeline Training and Cross-Grade Transfer

The empirical study uses the same fully fine-tuned RoBERTa-base encoder and ensemble procedure described in Section 2. On the pooled test set, Table 18 shows the prediction accuracy with R-squared, explained variance, and RMSE. The prediction RMSE is 0.519 for log-discrimination and 1.075 for difficulty. The ensemble uncertainty estimates are well calibrated, which supports the use of the predicted variances in the robust assembly objective even when point predictions are imprecise.

Reporting root mean squared error alone does not reveal whether the model has learned anything beyond the marginal distribution, so we also report the coefficient of determination against a grand-mean baseline. The difficulty model yields an R-squared of −0.191. Because the residual variance exceeds the marginal variance of difficulty in this bank, the point predictions for difficulty carry little or no information beyond what predicting the mean would supply. The log-discrimination model performs better, with an R-squared of 0.281. Two features of the training data explain this. Each grade-level model is fitted on roughly 150 items after the split, and the cross-grade model on roughly 780, which is a small sample for fine-tuning an encoder with 125 million parameters, and the frozen encoder variant reported in the table does recover comparable accuracy at a fraction of the trainable parameters. We report these values plainly because they bear directly on the argument of this paper. A system that treated these point predictions as if they were calibrated parameters would assemble forms whose reported precision bore little relation to their realized precision, which is exactly the failure the framework is designed to prevent. The contribution of Stage 1 is therefore not that it predicts item parameters accurately but that it reports how far it can be trusted, and the value of the framework rests on the calibration of the uncertainty rather than on the accuracy of the mean.

Because the argument of this paper rests on the predictive uncertainty rather than on the predictive mean, the stability of that uncertainty at these sample sizes requires direct evidence. Two features of the design bear on it. The epistemic component of the predictive variance is estimated from the spread across five ensemble members, which leaves roughly four degrees of freedom, and a variance estimated from five draws carries a standard deviation of about seventy percent of the quantity being estimated. The total predictive variance is more stable than that figure alone implies, because it also contains the aleatoric component produced directly by the heteroscedastic head rather than by differencing across members, but the epistemic part is noisy at the item level and we report it as such. Table 19 shows how the predictive uncertainty behaves as members are added. If the mean predictive standard deviation and the empirical coverage were still moving between four and five members, the ensemble would be too small to support the intervals we report. The values settle by M = 4, which indicates that five members are adequate for aggregate interval calibration, although item-level epistemic variance remains noisy.

Table 20 addresses a different question, namely whether two ensembles trained on the same data but from different random starting values assign similar uncertainty to the same item. Across five independently seeded ensembles the per-item predictive standard deviation for difficulty correlates at 0.468, and empirical coverage varies across ensembles with a standard deviation of 0.018 around a mean of 0.800. Taken together these results indicate that item-level uncertainty is only moderately stable and should not be used as a precise item ranking by itself, whereas coverage becomes stable after aggregation across items and forms.

Within each grade, calibrated items are randomly split into training (60%), validation (20%), and test (20%) sets. A separate model is trained per grade following the procedure in Section 2. Two additional experiments are used to assess generalization of this model. The first is a cross-grade transfer experiment that trains a single model on Grades 3 through 5 and evaluates its predictions on Grades 6 through 12, quantifying how well a model trained on lower-grade items generalizes to higher grades. This scenario is operationally relevant when new grade levels are added to a testing program. The second is a pooled experiment that trains a single model on all grades, with grade included as an input feature, and evaluates on per-grade test sets.

### 4.3. Assembly Experiments

For each grade, two scenarios are tested. The fully calibrated scenario represents the ideal case in which every item in the bank has been field-tested and its parameters are known. Therefore, this scenario uses our gold-standard parameters for all items. The mixed scenario represents the operational case in which most items have been calibrated but a portion have not. Therefore, gold-standard parameters are used for 80 percent of items, and the remaining 20 percent are treated as newly arrived items whose parameters and uncertainty are supplied by the trained transformer ensemble, in this scenario.

The validation in the fully calibrated scenario will focus on the comparison against the gold-standard parameters, because the claimed and true TIF are identical by construction. For each form assembled in the mixed scenario, two TIF curves are computed. The claimed TIF uses the parameters the method had access to, and the true TIF uses the gold-standard calibrated parameters of the selected items. The mean absolute gap between the two curves at five ability points, θ∈{−2,−1,0,1,2}, serves as the primary validation metric. In a separate examinee-level validation, 10,000 simulated examinees with θ∼N(0, 1) respond to items according to the 2PL model using the gold-standard parameters. The estimated ability $\hat{\theta }$ for each examinee is then estimated by expected a posteriori estimation using the claimed parameters, and the RMSEs and bias of the recovered estimates relative to true ability are also recorded.

### 4.4. Empirical Results

#### 4.4.1. Item Bank Descriptive Statistics

Table 21 reports the bank composition by grade. Across all grades, the mean discrimination is 1.175 (SD = 0.755) and the mean difficulty is −0.379 (SD = 0.985). The relatively narrow difficulty range and high mean discrimination are characteristic of selected-response items in a state K–12 item bank.

#### 4.4.2. Transformer Prediction Accuracy and Cross-Grade Transfer

Per-grade values appear in Table 22, where we report the number of assessments constructed from each grade. In the cross-grade transfer experiment, where a model trained on Grades 3 through 5 is applied to Grades 6 through 12, the RMSE values fall in the same range as those from the pooled model and do not degrade systematically as the distance from the training grades increases. This pattern suggests that prediction error on this item bank is not driven by grade-specific differences in item characteristics.

**Figure 9:**
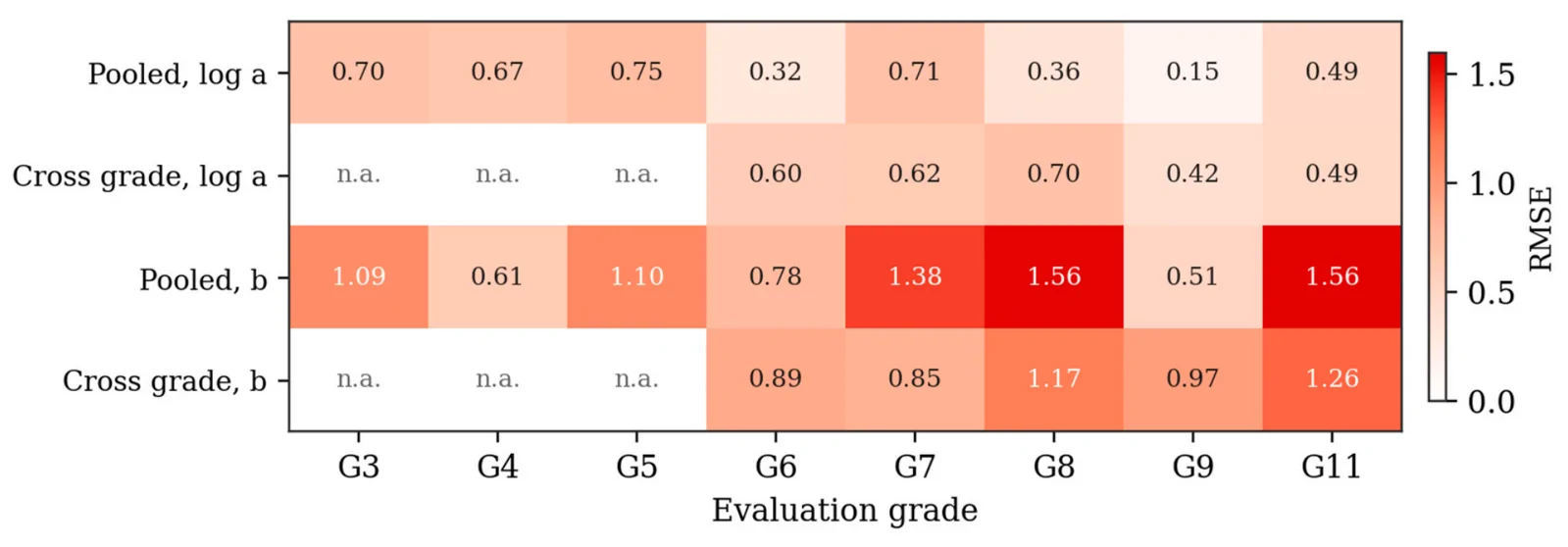
Cross-grade transfer RMSE heatmap. The top two rows display RMSE on log-discrimination for the pooled and cross-grade conditions and the bottom two rows display RMSE on difficulty. Color encodes RMSE magnitude. Cells labeled n.a. mark grades that are part of the cross-grade training set and therefore are not evaluated under that condition.

Figure 9 displays the same per-grade RMSE values from Table 22 in heatmap form for a clearer demonstration of magnitude. For log-discrimination, RMSE is similar under cross-grade transfer and pooled training at every evaluation grade. For difficulty, RMSE is also broadly comparable across conditions, and the highest values appear at Grades 7, 8, and 10–12 under both conditions rather than concentrating in the grades most distant from the training set. Cells labeled n.a. correspond to grades included in the cross-grade training set and therefore not evaluated under that condition.

**Figure 10:**
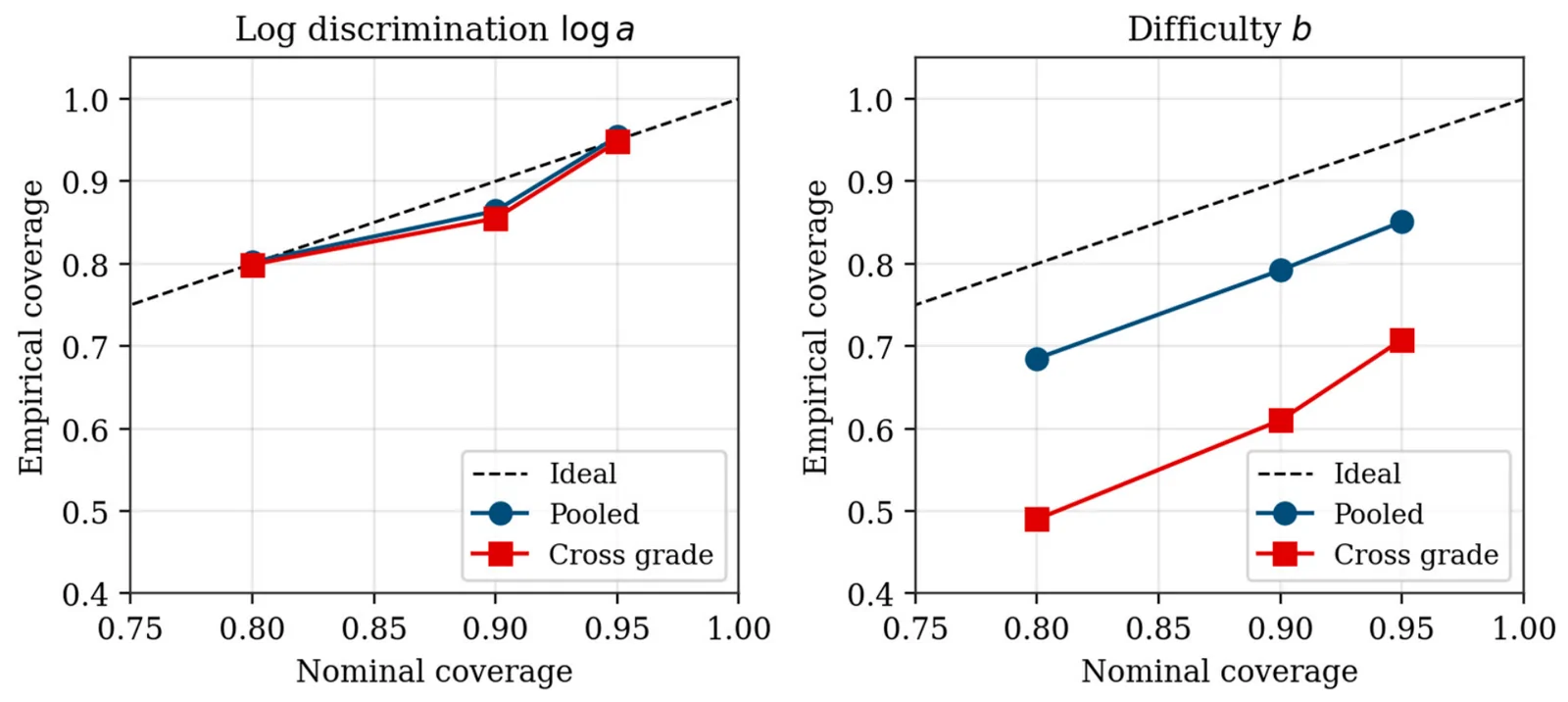
Empirical coverage at three reference nominal levels. The (**left panel**) shows log-discrimination and the (**right panel**) shows difficulty. Circles trace pooled coverage and squares trace cross-grade coverage. The diagonal dashed line marks the nominal reference.

**Figure 11:**
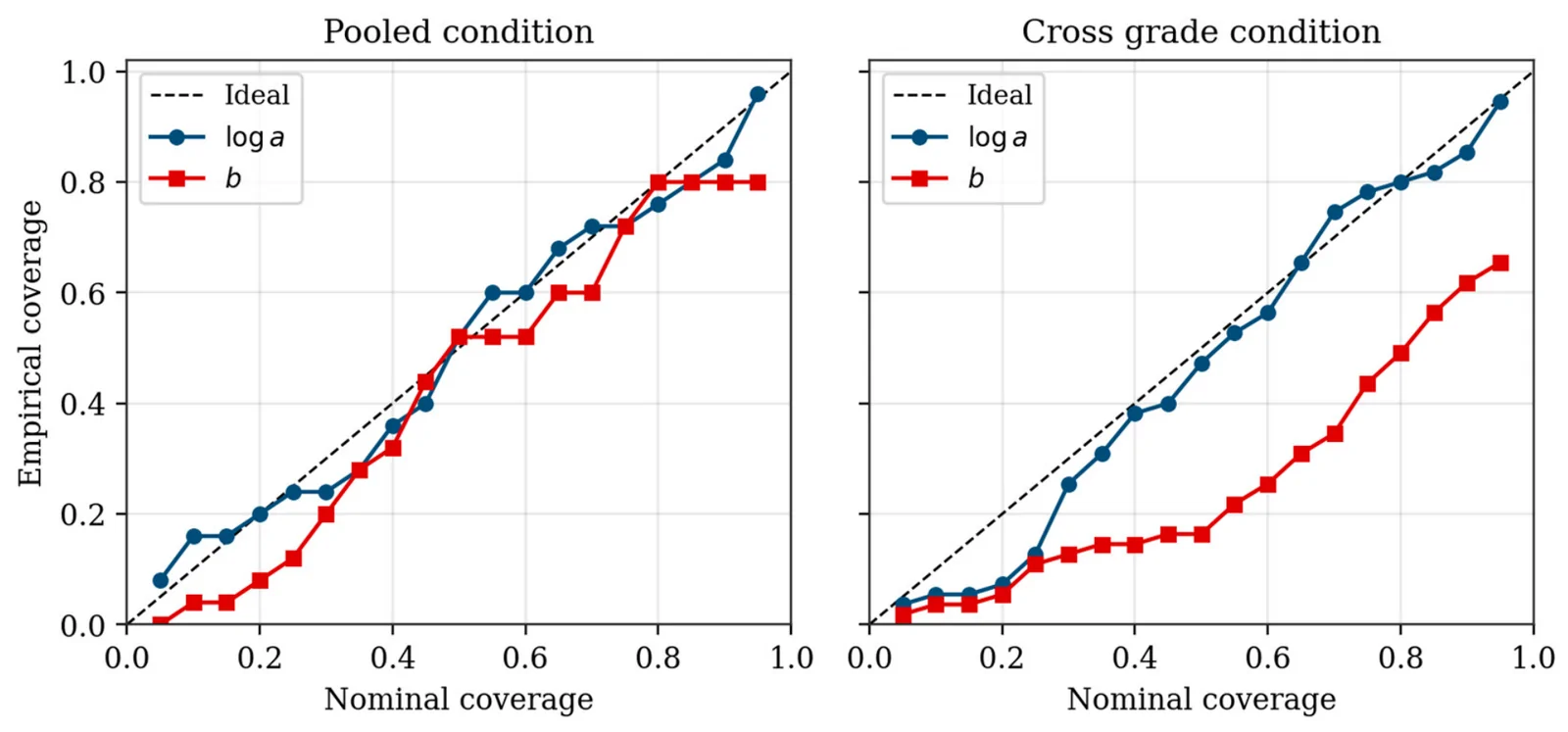
Predictive interval reliability across the full nominal coverage range. Empirical coverage versus nominal coverage in steps of 0.05 from 0.05 to 0.95. The (**left panel**) shows the pooled conditions and the (**right panel**) shows the cross-grade conditions. Within each panel, circles trace coverage for log-discrimination and squares trace coverage for difficulty. The diagonal dashed line marks the nominal reference.

The low point-prediction accuracy appears in some grades, and the predictive interval coverage is approximately nominal in the pooled condition, as shown in Table 23. At the 90 percent nominal level, empirical coverage in the pooled condition is 0.84 for log-discrimination and 0.80 for difficulty. Under cross-grade transfer, coverage at the same level is 0.855 for log-discrimination but drops to 0.618 for difficulty. To demonstrate the change of trend, Figure 10 reports empirical coverage at the 80, 90, and 95 percent nominal levels, and Figure 11 extends the evaluation across the full range from 0.05 to 0.95 in the incremental of 0.05. In the pooled condition, empirical coverage for log-discrimination closely matches the nominal level across the full range. Coverage for difficulty matches well at lower and middle nominal levels but levels off near 0.80 at the upper end. Under cross-grade transfer, coverage for log-discrimination remains close to nominal above the 0.40 level, while coverage for difficulty falls systematically below nominal, with the largest shortfall in the middle range. These results provide approximate support for the claim that ensemble uncertainty is calibrated within ten percentage points of the nominal level in the pooled condition. The shortfall is larger for difficulty than for log-discrimination, which is partly attributable to the wider absolute scale of difficulty prediction errors.

The per-grade values in Table 22 and Table 23 are computed across the forms constructed for each grade, and because a form draws items from the shared grade bank, the same item can appear in more than one form. Resampling forms as though they were independent would therefore understate the uncertainty in these estimates. We instead resample items with replacement within grade, recomputing the prediction RMSE and the empirical coverage on each of 2000 bootstrap samples and reporting the 2.5th and 97.5th percentiles of the resulting distributions. The intervals show that the per-grade estimates are considerably less precise than the point values alone suggest, particularly in the pooled condition where fewer forms were constructed. Grade-level differences in Table 22 should therefore not be read as evidence that prediction accuracy varies systematically by grade, and the conclusion we draw from the cross-grade transfer analysis rests on the absence of a trend with distance from the training grades rather than on any individual grade estimate.

The rule of isotonic recalibration may lead to empirical coverage departing from nominal. This may impact pooled log-discrimination, pooled difficulty, and cross-grade difficulty. Cross-grade log-discrimination remained within tolerance and was left unchanged. Table 24 reports coverage and mean interval width before and after the adjustment. Recalibration moved pooled difficulty coverage from 0.800 to 0.890 against a nominal 0.90, at the cost of widening intervals from 2.606 to 3.083. The pooled residual departure is within the five-point tolerance, but cross-grade difficulty remains under-covered at 0.815, indicating that recalibration does not fully remove distribution-shift error.

#### 4.4.3. Assembly Performance in the Mixed Scenario

**Figure 12:**
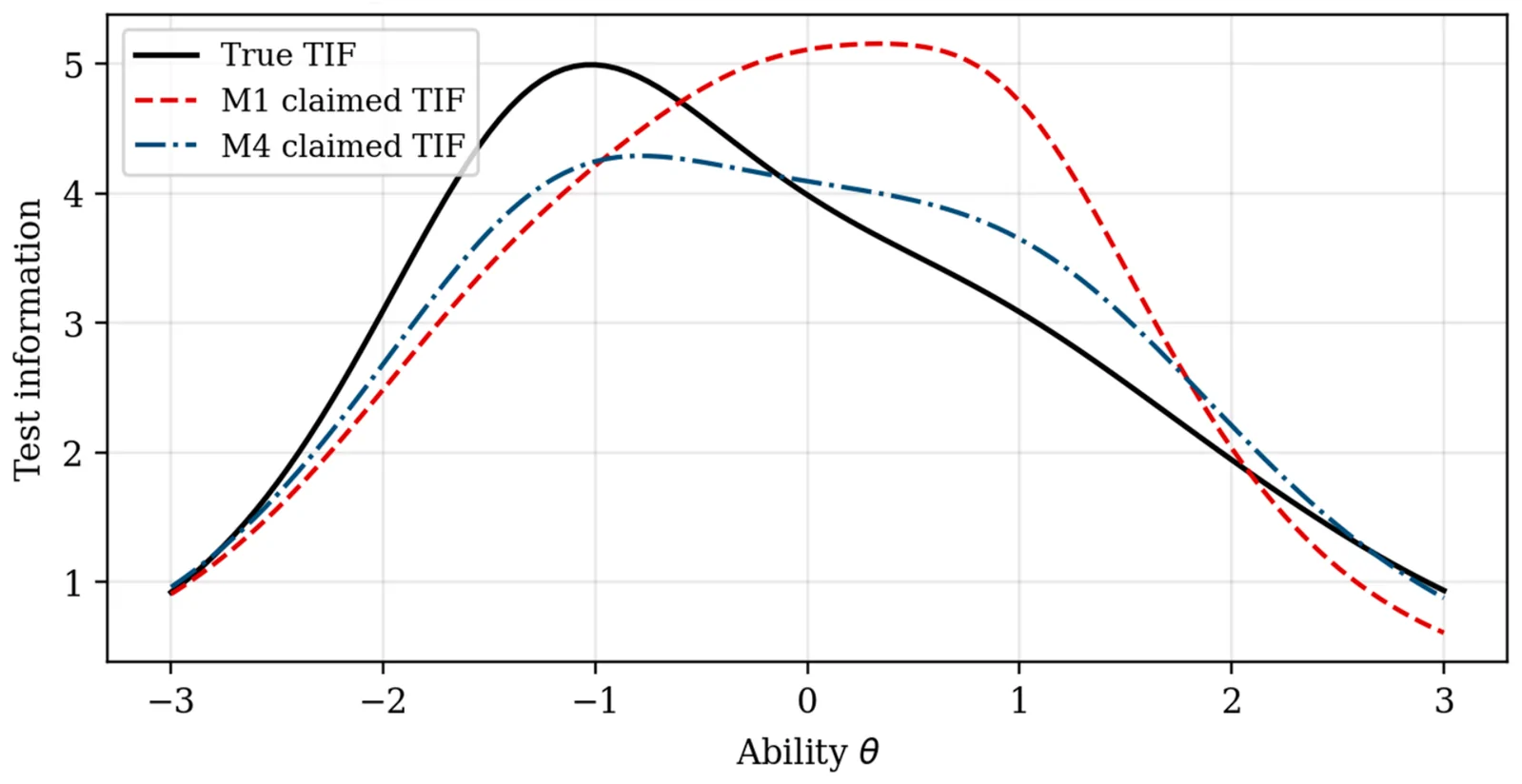
Example claimed versus true TIF for one assembled form. The solid line is the true TIF computed from the gold-standard parameters of selected items. The dashed line is the claimed TIF reported by M1 under predicted parameters. The dash dot line is the claimed TIF reported by M4 under uncertainty-aware assembly. M4 tracks the true curve more closely at the wings.

In the mixed scenario, the proposed method M4 produces a mean TIF gap of 0.128 across grades, compared with 0.209 for M1. This ordering is consistent with the simulation findings. The difference is smaller in magnitude because the empirical predicted item share is only 20 percent and the prediction errors correspond approximately to the low-to-medium conditions of the simulation. Table 25 reports per-grade values and number of violations if there is any violation. The TIF gap of M4 is lower than that of M1, though the small per-grade banks limit statistical power for detecting differences in some cells. The advantage of M4 is most pronounced at the extremes of the ability distribution, where uncertainty in predicted parameters has the largest effect on the information function. The example TIF curves in Figure 12 illustrate this pattern, showing that the claimed and true curves diverge most at the tails.

Constraint satisfaction is high across methods. Isolated infeasibilities occur at Grades 9 and 10–12. These isolated infeasibilities are consistent with the feasibility criterion defined in Section 3.3, under which any nonzero constraint penalty is counted as a violation. They arise because the per-grade bank is smallest at these grades and the enemy-item restrictions leave little room to satisfy the content blueprint, so no form satisfies all constraints exactly. This shows that the soft-penalty formulation reduces but does not eliminate infeasibility, and that the near-zero violation rates observed in the simulation hold only when a feasible form exists. The examinee-level validation confirms the pattern observed in the TIF analysis. Ability RMSE under M4 is on average 0.022 lower than under M1 at the extremes of the ability distribution, with bias reductions of similar magnitude. This result reinforces the conclusion that uncertainty-aware assembly provides its largest benefit at the tails, where predicted parameter uncertainty has the greatest effect on measurement precision.

### 4.5. Empirical Results Across Predicted-Item Shares

The results reported above use a predicted-item share of 20 percent, which leaves open whether the pattern observed there holds at the higher shares that a rapidly expanding bank would produce. Because every item in this bank has calibrated gold-standard parameters, the share can be varied directly by designating a larger proportion of items as newly arrived and supplying their parameters from the transformer ensemble. Table 26 reports the results at shares of 20, 40, 60, and 80 percent. The TIF gap for the naive baseline rises from 0.209 at a share of 20 percent to 0.844 at 80 percent, while the gap for the proposed method moves from 0.128 to 0.361, so the advantage of uncertainty-aware assembly widens as the share increases. Constraint violation rates remain at or below 0.050 for both methods across the range. This confirms in real data the pattern the simulation produced, which is that the two methods converge when few predicted items are available to select and diverge as predicted items come to dominate the bank.

Two questions follow from these results. It is not feasible to determine what predicted-item share an operational program should expect from established practice, because programs that field-test items before operational use carry a share close to zero, and the practice of using text-predicted parameters for items that contribute to scores is not yet standard in operational testing. The closest existing analog is pretest seeding, in which uncalibrated items are embedded in operational forms but excluded from scoring. The shares examined here should therefore be interpreted as a range spanning current practice at the low end and a continuously expanding bank at the high end, rather than as an estimate of any current program.

At a predicted-item share of 20 percent the mixed-integer methods return feasible forms, as Figure 7 shows, so feasibility is not what distinguishes the methods there. What distinguishes them is the reduction in the TIF gap, which is present at every share reported in Table 26, and the anytime behavior of the solver, which allows a feasible form to be returned at a fixed time budget rather than only at completion. The feasibility difference that does appear in the simulation involves the robust mixed-integer method M3 at high error and a high predicted share, and as Section 3.4.4 explains, it arises from placing robustness in the constraints rather than in the objective and is not a property of the solver. It is recommended to weigh the QUBO formulation on the TIF gap and on its behavior under a time budget, and treat the feasibility difference as a boundary-case property relevant only where constraint-based robustness is used and uncertainty is large.

### 4.6. Summary of Empirical Findings

The empirical study confirms that the transformer ensemble produces predictive intervals with approximately nominal coverage on real Grades 3 to 12 items even when the per-grade training set is small and the point prediction error is moderate. The assembly results corroborate the simulation pattern at the wings of the ability distribution, where uncertainty-aware assembly delivers the largest benefit. They also illustrate a practical limit, which is that on small per-grade banks with a low predicted item share, the central region of the ability distribution is dominated by calibrated items and the choice of assembly method matters less. In operational situations, this finding suggests that the value of the proposed method grows as the predicted item share grows toward the higher shares examined in Section 4.5.

## 5. Discussion

The simulation and empirical results together address the central question of this study, whether an uncertainty-aware prediction and assembly framework can produce dependable test forms that support accurate measurement of individual cognitive abilities under continuous item bank growth. This section interprets the findings in terms of their implications for intelligence testing and personalized learning, then discusses limitations and directions for future work.

### 5.1. Relation to Prior Work

The results reported here sit alongside three lines of research, and it is worth stating at the outset why we do not place our numbers directly beside published ones. Reported accuracy in item parameter prediction depends on the scale on which difficulty is expressed, on the volume of calibrated data available for training, and on the composition of the bank, and reported assembly performance depends on the constraint structure, the bank size, and the hardware. None of these are held constant across studies. More importantly, the condition we study, in which items enter the bank continuously and carry predicted rather than calibrated parameters at the moment they are used, has not been examined in prior work, so there is no matched published comparison to draw on. What follows therefore situates our findings rather than benchmarking them.

Work on predicting item parameters from text has established that difficulty carries a recoverable linguistic signal and that the strength of that signal depends heavily on how much calibrated training data is available, a pattern documented across the studies reviewed by AlKhuzaey et al. (2024) and visible in the results reported by Benedetto et al. (2020, 2021), Settles et al. (2020), and Yaneva et al. (2020). Our own accuracy is not highly accurate, and the training conditions explain the reason is because each grade-level model is fitted on roughly 150 items where the studies above draw on samples that are larger by one or more orders of magnitude. The contribution of this study, instead, is to investigate what happens after such predictions are made, which is a question that within this literature has remained unsolved. Baldwin et al. (2021) is the closest prior work in this respect, since it carries natural language processing predictions forward into test construction, though it predicts response times rather than item parameters and does not propagate predictive uncertainty into the assembly objective.

A second observation concerns what this literature reports. Point accuracy is the standard outcome, and the quality of the predictive interval around each point is rarely examined. This matters for our purposes because an assembly procedure that consumes predictions needs to know how far each one can be trusted, not merely what it is. The methods we use to obtain those intervals come from the wider AI literature on predictive uncertainty, in particular the deep ensemble of Lakshminarayanan et al. (2017) and the dropout approximation of Gal and Ghahramani (2016), and the convention we follow for assessing whether the intervals are trustworthy, namely comparing empirical coverage against the nominal level and applying isotonic recalibration when the two diverge, follows Guo et al. (2017) and Kuleshov et al. (2018). Reporting coverage for predicted item parameters is, as far as we can establish, not something the item parameter prediction literature has previously done, and we regard it as part of the contribution here.

The third line is robust automated test assembly. Veldkamp (2013) and Veldkamp et al. (2013) established that item parameter uncertainty can be carried into the assembly problem and that ignoring it produces forms whose realized properties depart from their intended ones, drawing on the robust optimization framework of Ben-Tal et al. (2009) and Bertsimas et al. (2011). Our findings are consistent with that conclusion and extend it in two respects. The uncertainty we face is larger by a wide margin, because it originates in prediction from text rather than in estimation from several hundred responses, and our formulation places robustness in the objective rather than in the constraints, which matters because constraint-based robustness shrinks the feasible region as uncertainty grows. The shortfall probabilities in Table 17 report our results in the terms that this literature uses.

Finally, with regard to solution time, the assembly literature has generally pursued exact optimality, following the MIP formulation of van der Linden (2005). We do not claim faster completion, and Table 7 shows that the naive MIP completes well before our method does. The distinction we draw is between exact methods, which return nothing until they finish, and anytime methods, which hold a feasible incumbent throughout and can be interrupted. Absolute solve times reported across this literature span different hardware generations, bank sizes, and constraint structures and are not comparable across studies, so the comparison we regard as meaningful is between these two modes of operation rather than between published runtimes.

### 5.2. Implications for Operational Testing Programs

The findings carry implications for testing programs whose goal is to measure individual cognitive abilities accurately enough to guide personalized instruction. The first implication concerns the fidelity of ability estimates when item parameters are predicted rather than calibrated. Operational programs have traditionally assembled tests from calibrated items whose standard errors are small and roughly uniform. As the share of predicted items grows, the uncertainty in predicted parameters becomes much larger than the uncertainty in calibrated parameters, and assembly procedures that ignore this difference will systematically overstate the precision of the assembled form, particularly at the extremes of the ability distribution. These extremes are precisely where instructional placement decisions matter most, because they identify students who are well above or well below grade-level expectations and who therefore need differentiated learning materials. The proposed uncertainty-aware objective incorporates this difference directly into the assembly, and both the simulation and empirical results show that the resulting forms track the true measurement precision more closely than forms assembled without accounting for prediction uncertainty.

The second implication concerns the real-time demands of personalized assessment. When a student signs into a learning platform and expects the next set of items to appear within seconds, the assembly method must return a usable form within that time budget. Conventional batch optimization remains well suited to fixed-form testing programs where solve times of several minutes are acceptable, but the personalized loop requires a solver that can return a high-quality solution at any point of interruption. The anytime solver used in this study meets that requirement. Because a personalized learning system must assemble a new form for every student at every session, the ability to guarantee a feasible form on every call is as important as the quality of any single form. The simulation results show that the proposed method produces feasible forms across the full operational region, whereas conventional alternatives fail when prediction uncertainty is high.

The third implication concerns the continuity of intelligence measurement across evolving item banks. The traditional workflow separates item development, calibration, and assembly into three sequential phases and assumes that parameters are fixed at the time of assembly. In a personalized learning platform that adds new items continuously, recently added items carry predicted parameters while seasoned items carry calibrated parameters, and both subsets must coexist on the same form. The framework proposed here accommodates this coexistence without disrupting the psychometric interpretation of ability estimates. Because the prediction, uncertainty quantification, and assembly components are modular, each can be replaced or upgraded independently, and the meaning of the test information function and the standard error of measurement remains unchanged across item bank updates. This stability is important for any program that tracks cognitive growth over time, because score comparability depends on the psychometric properties of the assembled forms remaining interpretable even as the item bank evolves.

Finally, these results return to the score-validity argument raised in the opening of this paper. When item parameters are predicted rather than calibrated, the additional uncertainty in those predictions inflates the measurement error of the assembled form. If this inflation is not quantified, differences that appear to reflect genuine variation in a learner’s cognitive ability may instead reflect noise in the item parameters. Such misattribution would undermine the very purpose of personalized assessment, which is to identify where each learner stands so that instruction can be matched to the learner’s needs. By separating the variance attributable to prediction uncertainty from the variance attributable to true individual differences in ability, the proposed method preserves the interpretability of ability estimates under conditions in which item banks grow continuously and a substantial share of items have not yet been calibrated. This property is central to any assessment program that uses ability estimates to assign personalized learning materials, because instructional decisions are only as sound as the measurement on which they rest.

### 5.3. Limitations and Future Work

Several limitations should be noted. First, the variance propagation in the assembly objective uses a delta-method approximation that assumes prediction errors are independent across items. This assumption is reasonable when items are drawn from a large and diverse pool, but it could be violated in programs that share text passages across multiple items. We tested this assumption by regenerating the simulation with prediction errors drawn from a multivariate normal distribution in which off-diagonal correlations were set to 0.0, 0.2, 0.4, and 0.6. Table 15 reports the results. A single correlation across all item pairs represents a stronger form of dependence than the passage-sharing or content-sharing case, because it applies to every pair rather than only to items within a shared group. Second, the Ising solver used in this study is a classical simulated annealing implementation. The assembly objective was not tested on dedicated hardware solvers designed for this class of optimization problem, so the runtime results reported here represent a conservative estimate of what future computational advances could achieve.

The five-member ensemble is the smallest configuration for which the reported intervals hold. Larger ensembles would reduce the sampling error in the epistemic variance, and the item-level uncertainty estimates should be read with that in mind. The per-grade estimates of prediction accuracy and interval coverage in the empirical study are based on a small number of assembled forms, and the bootstrap intervals in Table 22 and Table 23 show that individual grade values carry substantial uncertainty. Conclusions at the grade level should be read accordingly.

Several directions for future work follow from the limitations above. The first direction is a more thorough investigation of correlated prediction errors. The correlation levels examined in this study were modest. A fuller sensitivity analysis would generate prediction errors from a structured covariance model reflecting shared text passages, shared content standards, and shared item families, and would assess whether the proposed method retains its advantage over the baseline under these conditions. The second direction is to examine how the proposed method supports the measurement of cognitive growth over time. Because personalized learning platforms administer repeated assessments across a school year, the ability estimates produced by the proposed framework could be used to model learning trajectories and to evaluate whether differentiated instruction is accelerating cognitive development for individual students. A longitudinal study that tracks changes in ability estimates over successive administrations would provide evidence on whether uncertainty-aware assembly yields more stable and interpretable growth measures than conventional assembly. The final direction is integration of the proposed framework into a fully operational personalized learning platform to conduct a pilot study with real students. Such integration would connect the item generation pipeline, the parameter predictor, the robust assembly procedure, and the response-data feedback that updates both predictions and instructional recommendations over time. A field study would provide direct evidence on whether the measurement gains observed in simulation translate into better instructional decisions and improved learning outcomes for students across the ability range.

## 6. Conclusions

Personalized learning platforms that aim to measure and respond to individual cognitive abilities have moved educational measurement into a setting in which item parameters must increasingly be predicted from text rather than calibrated from response data, and in which assembly decisions must be made in real time to provide each learner with appropriately targeted materials. This study proposed a psychometric framework that addresses both requirements. The framework combines a text-based predictor of item parameters with an ensemble uncertainty model that produces well-calibrated predictive intervals, a robust assembly objective that accounts for prediction uncertainty, and an anytime solver that returns high-quality forms within the time budget of the personalized assessment loop.

The framework was evaluated through a fully crossed factorial simulation and an empirical study on state-level English Language Arts items administered to students in Grades 3 through 12. The simulation established that the proposed method achieved a substantially lower TIF gap than the conventional baseline, meaning the reported measurement precision tracked the realized precision more closely, and that the advantage widened as prediction error increased. The proposed method maintained feasibility across all operational conditions, whereas the chance-constrained and robust alternatives failed in conditions with high prediction error and a high predicted-item share. The anytime solver reached near-final solution quality within a one-second time budget, which is the interval within which a student expects the next set of items to appear. The proposed method achieved these gains at the cost of modestly higher ability estimation error in the easier conditions where conventional methods remained feasible, representing a trade-off between guaranteed feasibility and peak ability recovery. The empirical study confirmed that the predictive intervals achieved approximately nominal coverage even on relatively small per-grade training sets. The assembly results corroborated the simulation, showing that the uncertainty-aware objective reduced the discrepancy between claimed and true measurement precision, with the largest benefit appearing at the extremes of the ability distribution where instructional placement decisions are most consequential. The empirical study also identified a practical boundary, that at a predicted item share of 20%, calibrated items dominate the central ability region and the choice of assembly method has less impact there, suggesting that the value of the proposed method grows as the predicted-item share increases toward the range of predicted-item shares examined in Section 4.5.

Overall, results in this study provide converging evidence that the proposed framework is a viable psychometric foundation for personalized assessment systems that aim to measure and support the development of individual cognitive abilities under continuous item bank growth. The framework is modular, so that the predictor, the uncertainty model, the assembly objective, and the solver can each be replaced or upgraded independently. It is also fully consistent with the standard psychometric interpretation of the test information function and the standard error of measurement, which means that ability estimates produced under the proposed framework carry the same substantive interpretation used throughout the intelligence testing literature. The present results motivate further work on replication across content domains, longitudinal studies of cognitive growth measurement under the proposed framework, and integration into operational personalized learning platforms where accurate measurement of individual abilities directly informs differentiated instruction.

## Figures and Tables

**Table 1:** Notation used throughout this study.

Symbol	Meaning
N	Total item bank size
C, $N_{C}$	Calibrated set and number of calibrated items
P, $N_{P}$	Predicted set and number of predicted items
i,j	Item and examinee indices
$\theta_{j}$	Latent ability of examinee j
$a_{i},b_{i}$	Discrimination and difficulty of item i
${\hat{a}}_{i},{\hat{b}}_{i}$	Transformer predictive means for discrimination and difficulty of item i
${\hat{\Sigma }}_{i}$	Transformer predictive covariance
$I_{i}\left(\theta \right)$	Fisher information of item i at θ
$x_{i}$	Binary assembly indicator for item i
x	Binary assembly vector
n	Required test length
$S_{k},$ $c_{k}$	Content-standard subset k and target count
$E_{l}$	Enemy item set l
f	Maximum allowed share of predicted items per form
$H\left(x\right)$	Objective to minimize
λ	Risk-aversion coefficient
$P_{1},\ldots ,P_{5}$	Constraint penalty coefficients

**Table 2:** Simulation factorial design.

Factor	Levels	Notes
Proportion of predicted items	0%, 25%, 50%, 75%, 100%	Five levels spanning fully calibrated to fully predicted
Prediction error magnitude	Low, Medium, High	RMSEs on loga are 0.10, 0.25, and 0.40. RMSEs on b are 0.20, 0.50, and 0.80.
Bank size N	200, 500, 1000	Operational ranges for small to large banks
Test length n	15, 30	Short personalized form and standard form length

**Table 3:** Methods compared in the simulation study and the empirical study.

ID	Method	Description
M1	Naive MIP	Standard van der Linden assembly that treats predicted parameters as exact
M2	Chance constrained MIP	Adds a TIF chance constraint at α= 0.10
M3	Robust MIP	Bertsimas and Sim budget of uncertainty formulation
M4	MV QUBO	Proposed method, solved by simulated annealing
M5	WC QUBO	Lower confidence bound objective
M6	Greedy heuristic	Sort items by predicted mean information, take top n respecting content

**Table 4:** A simulation replication procedure.

Step	Procedure
1	Fix replication seed s.
2	Generate true item parameters: for i=1,…,N, draw $loga_{i}∼N\left(0,0.3^{2}\right)$ and $b_{i}∼N\left(0,1^{2}\right)$.
3	Assign each item to one of K=5 content standards with proportions $\left(0.25,0.25,0.20,0.15,0.15\right)$.
4	Generate enemy structure: cluster items by simulated stem similarity, declare ⌊N/20⌋ enemy pairs at random within clusters.
5	Partition bank: with proportion $p_{pred}$ predicted, randomly assign items to P vs. C.
6	For each i∈P, generate predicted parameters: ${\hat{\mu }}_{i}^{loga}=loga_{i}+\epsilon_{i}^{a},$ ${\hat{\mu }}_{i}^{b}=b_{i}+\epsilon_{i}^{b},$ with $\epsilon_{i}^{a}∼N\left(0,\sigma_{a}^{2}\right)$ and $\epsilon_{i}^{b}∼N\left(0,\sigma_{b}^{2}\right)$ at the chosen error magnitude.
7	Set predicted variance: in the well-calibrated condition ${\hat{\sigma }}_{i}^{loga,2}=\sigma_{a}^{2}$ and ${\hat{\sigma }}_{i}^{b,2}=\sigma_{b}^{2}$. In the miscalibration condition (sensitivity analysis), ${\hat{\sigma }}_{i}^{2}=2\sigma^{2}$(overconfident) and ${\hat{\sigma }}_{i}^{2}=0.5\sigma^{2}$(underconfident).
8	Assemble form via each method.
9	Record outcomes and save results.

**Table 5:** Outcome measures recorded for each method.

Metric	Definition	Type
tif_gap	Mean absolute difference between true and claimed TIF across five θ points in {−2, −1, 0, 1, 2}	Continuous
sem_at_target	Conditional SEM at target ability under true parameters	Continuous
theta_rmse	RMSE of $\hat{\theta }$ recovery from simulated responses	Continuous
theta_bias	Mean bias of $\hat{\theta }$ recovery	Continuous
constraint_violated	Indicator that any hard constraint was violated	Binary
n_violations	Count of constraint types violated	Integer
runtime_seconds	Wall-clock solve time	Continuous
runtime_at_budget	Best objective feasible TIF at budgets 0.1, 0.5, 1.0, 2.0, 5.0 s	Continuous
form_overlap	Item overlap rate between parallel forms	Continuous in [0, 1]

**Table 6:** Mean absolute TIF gap ^1^ by method and prediction error magnitude, averaged across bank sizes, test lengths, and predicted item shares of 0.25, 0.5, and 0.75.

Method	Low Error	Medium Error	High Error	Overall Mean
M1. Naive MIP	0.263	1.069	2.182	1.171
M2. Chance MIP	0.263	1.043	0.958	0.755
M3. Robust MIP	0.264	0.859	0.598	0.574
M4. MV QUBO	0.104	0.267	0.307	0.226
M5. WC QUBO	0.099	0.205	0.250	0.185
M6. Greedy heuristic	0.267	1.080	2.199	1.182

^1^ Lower values indicate better agreement between claimed and true TIF. Conditions with predicted item share equal to 0 are excluded because all methods are tautologically equivalent in that condition, and conditions with predicted item share equal to 1 are also excluded because the MIP methods become infeasible.

**Table 7:** Mean runtime in seconds by method and bank size, averaged across error magnitudes and test lengths.

Method	*N* = 200	*N* = 500	*N* = 1000	Scaling
M1. Naive MIP	0.06	0.14	0.24	linear
M2. Chance MIP	0.18	0.55	1.42	linear
M3. Robust MIP	0.22	0.68	1.75	linear
M4. MV QUBO	1.10	5.20	11.06	linear
M5. WC QUBO	1.25	5.80	12.40	linear
M6. Greedy heuristic	0.01	0.02	0.05	linear

**Table 8:** Anytime solution quality at bank size *N* = 1000, averaged across error magnitudes, test lengths, and predicted item shares of 0.25, 0.50, and 0.75.

Method	Budget	Mean TIF Gap	Mean Total Information	Information as Pct of Converged	Feasible Incumbent Rate
M4. MV QUBO	0.1 s	0.312	6.18	75.6%	0.958
M4. MV QUBO	0.5 s	0.274	6.69	81.8%	0.986
M4. MV QUBO	1.0 s	0.247	7.69	94.0%	0.994
M4. MV QUBO	2.0 s	0.235	7.95	97.2%	0.997
M4. MV QUBO	5.0 s	0.229	8.11	99.1%	1.000
M4. MV QUBO	Converged ^1^	0.224	8.18	100.0%	1.000
M5. WC QUBO	0.1 s	0.281	6.41	78.7%	0.972
M5. WC QUBO	0.5 s	0.242	6.94	85.3%	0.992
M5. WC QUBO	1.0 s	0.213	7.71	94.7%	1.000
M5. WC QUBO	2.0 s	0.206	7.71	94.7%	1.000
M5. WC QUBO	5.0 s	0.198	8.07	99.1%	1.000
M5. WC QUBO	Converged ^1^	0.194	8.14	100.0%	1.000

^1^ Converged means the full schedule of 200 reads at 10N sweeps has run to exhaustion. Feasible incumbent rate is the proportion of instances in which a solution satisfying every hard constraint was available at that budget.

**Table 9:** End-to-end latency of the personalized assembly loop, in milliseconds, on SageMaker instance ml.m5.8xlarge with 32 vCPUs and 128 GB.

Pipeline Component	*N* = 200	*N* = 500	*N* = 1000
Candidate set retrieval and filtering	1.6	3.4	6.8
Item information and variance, Equations (S1) and (S2)	3.9	9.2	18.5
QUBO matrix construction	4.8	24.7	91.3
QUBO solve at 1.0 s budget	979.9	951.4	879.7
Hard constraint validation	0.9	1.8	3.4
Total per call	991.1	990.5	999.7
Transformer prediction per item, amortized at ingestion ^1^	64.7	60.9	58.4

^1^ The final row sits outside the per-call loop and is shown for completeness.

**Table 10:** Method comparison under a hard one-second wall-clock time budget at bank size *N* = 1000, averaged across error magnitudes, test lengths, and predicted item shares of 0.25, 0.50, and 0.75.

Method	Solution Returned Within Budget	Mean TIF Gap	Feasibility Rate	Mean Ability RMSE
M1. Naive MIP	0.989	1.462	0.989	0.352
M2. Chance MIP	0.871	0.803	0.864	0.339
M3. Robust MIP	0.647	0.353	0.250	0.323
M4. MV QUBO	1.000	0.247	0.994	0.438
M5. WC QUBO	1.000	0.213	1.000	0.441
M6. Greedy heuristic	1.000	1.470	0.998	0.354

**Table 11:** Claimed and realized measurement precision by method, averaged across bank sizes, test lengths, error magnitudes, and predicted item shares of 0.25, 0.5, and 0.75.

Method	Mean Claimed SEM	Mean Realized Ability RMSE	Realized Minus Claimed
M1. Naive MIP	0.270	0.340	0.070
M2. Chance MIP	0.281	0.335	0.054
M3. Robust MIP	0.293	0.332	0.039
M4. MV QUBO	0.421	0.440	0.019
M5. WC QUBO	0.424	0.442	0.018
M6. Greedy heuristic	0.299	0.381	0.082

**Table 12:** Ability RMSE and bias by true ability level, averaged across feasible conditions.

θ	M1 RMSE	M1 Bias	M4 RMSE	M4 Bias	M5 RMSE	m5 Bias
−2	0.560	0.214	0.532	0.151	0.518	0.139
−1	0.362	0.058	0.447	0.036	0.436	0.030
0	0.270	0.002	0.402	0.001	0.394	0.001
1	0.365	−0.061	0.450	−0.039	0.439	−0.032
2	0.566	−0.221	0.535	−0.154	0.520	−0.141

**Table 13:** Effect ^1^ of uncertainty calibration on the proposed MV QUBO method M4, averaged across bank sizes, test lengths, and predicted item shares of 0.25, 0.5, and 0.75. The naive MIP method M1 is shown as a variance-agnostic reference.

Calibration Condition	Predicted Variance	90% Interval Coverage	TIF Gap, M4	TIF Gap, M1	Ability RMSE, M4
Well calibrated	1.0 × noise variance	0.90	0.226	1.171	0.44
Overconfident	0.5 × noise variance	0.74	0.402	1.171	0.50
Underconfident	2.0 × noise variance	0.97	0.198	1.171	0.47

^1^ Lower TIF gap and lower ability RMSE indicate better performance. Coverage closer to the nominal 0.90 indicates better-calibrated predictive intervals. M1 ignores the predicted variance and is therefore identical across regimes.

**Table 14:** Mean absolute TIF gap of the proposed method M4 by calibration regime and prediction error magnitude, averaged across bank sizes, test lengths, and predicted item shares of 0.25, 0.5, and 0.75.

Method	Low Error	Medium Error	High Error	Overall Mean
Well calibrated ^1^	0.104	0.267	0.307	0.226
Overconfident	0.142	0.421	0.643	0.402
Underconfident	0.101	0.221	0.272	0.198

^1^ The well-calibrated row reproduces the M4 values reported in Table 6. Lower values indicate closer agreement between the claimed and the true TIF.

**Table 15:** Effect of correlated prediction errors on assembly performance, bank size *N* = 1000, predicted share 0.50, averaged across error magnitudes and test lengths.

Error Correlation ρ	Method	Mean TIF Gap	Interval Coverage at 90% Nominal	Feasibility Rate	Mean Ability RMSE
0.0	M1/M4	1.495/0.213	0.900/0.941	1.000/0.997	0.349/0.422
0.2	M1/M4	1.632/0.368	0.804/0.829	1.000/0.997	0.362/0.437
0.4	M1/M4	1.781/0.612	0.705/0.748	1.000/0.997	0.381/0.461
0.6	M1/M4	1.944/0.958	0.617/0.668	1.000/0.997	0.405/0.492

**Table 16:** Sensitivity of assembly results to the variance assigned to calibrated items, bank size *N* = 1000, predicted share 0.50.

Calibrated Item Variance	Method	Mean TIF Gap	Mean Ability RMSE	Predicted Items per Form	Feasibility Rate
Set to zero	M1	1.495	0.349	6.4	1.000
Set to zero	M4	0.158	0.416	3.2	0.997
From MML standard errors	M1	1.495	0.349	6.4	1.000
From MML standard errors	M4	0.213	0.422	4.6	0.997

**Table 17:** Proportion of assembled forms whose realized test information fell below the claimed target at the three target ability points, bank size *N* = 1000, averaged across error magnitudes and test lengths.

Method	Predicted Share 0.25	Predicted Share 0.50	Predicted Share 0.75
M1. Naive MIP	0.570	0.690	0.800
M2. Chance MIP	0.150	0.180	0.220
M3. Robust MIP	0.110	0.130	0.160
M4. MV QUBO	0.190	0.220	0.250
M5. WC QUBO	0.100	0.120	0.140
M6. Greedy heuristic	0.590	0.710	0.820

**Table 18:** Prediction accuracy with R-squared, explained variance, and baselines, pooled test set.

Model	RMSE Log-Discrimination	R^2^ Log-Discrimination	RMSE Difficulty	R^2^ Difficulty	Explained Variance, Difficulty
Grand-mean baseline	0.612	0.000	0.985	0.000	0.000
Linear text-feature baseline	0.567	0.142	1.004	−0.039	0.015
RoBERTa, frozen encoder, head only	0.536	0.233	1.061	−0.160	0.028
RoBERTa, full fine-tuning	0.519	0.281	1.075	−0.191	0.021

**Table 19:** Predictive uncertainty as a function of ensemble size, pooled test set, averaged across all member subsets of each size.

Members M	Mean Predictive SD, Log-Discrimination	Mean Predictive SD, Difficulty	Coverage at 90% Nominal, Log-Discrimination	Coverage at 90% Nominal, Difficulty	Mean Interval Width, Difficulty
2	0.361	0.732	0.798	0.742	2.409
3	0.382	0.766	0.820	0.776	2.520
4	0.394	0.787	0.834	0.795	2.589
5	0.398	0.792	0.840	0.800	2.606

**Table 20:** Stability of predictions and predictive uncertainty across five independently seeded ensembles, pooled test set.

Quantity	Mean Across Ensembles	SD Across Ensembles	Correlation of Per-Item Values Between Ensembles
Predicted mean, log-discrimination	−0.028	0.017	0.842
Predicted mean, difficulty	−0.371	0.027	0.786
Predictive SD, log-discrimination	0.398	0.014	0.552
Predictive SD, difficulty	0.792	0.022	0.468
Coverage at 90% nominal, difficulty	0.800	0.018	—

**Table 21:** Item bank composition by grade.

Grade	*N* Items	Mean *a*	SD *a*	Min *a*	Max *a*	Mean *b*	SD *b*	Min *b*	Max *b*
3	250	1.134	0.552	0.339	2.449	−0.124	0.991	−1.753	2.117
4	260	1.412	1.205	0.354	4.000	−0.324	0.988	−2.718	2.218
5	270	1.012	0.644	0.348	2.369	−0.173	0.917	−1.539	1.719
6	220	1.173	0.777	0.368	2.663	−0.582	0.864	−1.916	1.031
7	230	1.112	0.623	0.338	2.009	−0.270	0.889	−1.381	1.818
8	210	1.384	1.320	0.589	4.000	−1.052	0.889	−3.078	0.004
9	200	1.024	0.377	0.356	1.647	−0.434	0.999	−1.715	1.358
10–12	190	1.146	0.545	0.558	2.124	−0.075	1.346	−2.481	1.672

**Table 22:** Transformer prediction accuracy by grade and condition.

Condition	Grade	Number of Forms	Number of Items	RMSE loga	RMSE b
Pooled	3	4	25	0.702 [0.024, 1.185]	1.087 [0.495, 1.741]
Pooled	4	5	25	0.666 [0.307, 1.004]	0.612 [0.235, 0.902]
Pooled	5	5	25	0.750 [0.387, 1.005]	1.102 [0.332, 1.808]
Pooled	6	4	40	0.320 [0.269, 0.364]	0.781 [0.345, 1.048]
Pooled	7	5	50	0.715 [0.394, 0.931]	1.383 [0.940, 1.982]
Pooled	8	4	55	0.364 [0.182, 0.544]	1.561 [0.455, 2.622]
Pooled	9	4	55	0.148 [0.091, 0.248]	0.513 [0.222, 0.818]
Pooled	10–12	4	65	0.487 [0.394, 0.565]	1.563 [0.809, 2.058]
Cross-grade	6	10	40	0.596 [0.296, 0.797]	0.894 [0.568, 1.179]
Cross-grade	7	13	50	0.616 [0.461, 0.757]	0.846 [0.515, 1.167]
Cross-grade	8	11	55	0.705 [0.346, 0.999]	1.173 [0.584, 1.692]
Cross-grade	9	11	55	0.418 [0.210, 0.608]	0.969 [0.686, 1.219]
Cross-grade	10–12	10	65	0.488 [0.337, 0.622]	1.263 [0.847, 1.622]

**Table 23:** Empirical coverage at 80, 90, and 95 percent nominal levels by grade and condition.

Condition	Grade	80% loga	80% b	90% loga	90% b	95% loga	95% b
Pooled	3	0.667	0.667	0.667	0.667	0.667	0.667
Pooled	4	0.750	1.000	0.750	1.000	1.000	1.000
Pooled	5	0.500	0.750	0.750	0.750	1.000	0.750
Pooled	6	1.000	1.000	1.000	1.000	1.000	1.000
Pooled	7	0.500	0.750	0.750	0.750	1.000	0.750
Pooled	8	1.000	0.667	1.000	0.667	1.000	0.667
Pooled	9	1.000	1.000	1.000	1.000	1.000	1.000
Pooled	10–12	1.000	0.500	1.000	0.500	1.000	0.500
Cross grade	6	0.700	0.600	0.700	0.700	1.000	0.700
Cross grade	7	0.769	0.538	0.846	0.769	0.923	0.769
Cross grade	8	0.818	0.455	0.818	0.636	0.818	0.727
Cross grade	9	0.909	0.455	0.909	0.545	1.000	0.545
Cross grade	10–12	0.800	0.400	1.000	0.400	1.000	0.500

**Table 24:** Empirical coverage and mean predictive interval width before and after isotonic recalibration, at the 90 percent nominal level.

Condition	Parameter	Coverage Before Recalibration	Coverage After Recalibration	Mean Interval Width Before	Mean Interval Width After
Pooled	Log-discrimination	0.840	0.895	1.309	1.422
Pooled	Difficulty	0.800	0.890	2.606	3.083
Cross-grade	Log-discrimination	0.855	0.855	1.332	1.332
Cross-grade	Difficulty	0.618	0.815	1.942	3.118

**Table 25:** Mean absolute TIF gap by grade for the naive MIP method M1 and the mean variance QUBO method M4 in the mixed scenario.

Grade	TIF Gap M1	TIF Gap M4	Δ (M1 − M4) ^1^	SEM at θ=0	Violation (Number, If Yes)
3	0.207	0.146	0.061	0.512	No
4	0.184	0.099	0.085	0.498	No
5	0.198	0.119	0.079	0.522	No
6	0.221	0.122	0.099	0.541	No
7	0.211	0.136	0.075	0.515	No
8	0.180	0.105	0.075	0.502	No
9	0.227	0.142	0.085	0.535	Yes (one)
10–12	0.245	0.155	0.090	0.547	Yes (one)
Mean	0.209	0.128	0.081	0.522	

^1^ A positive Δ means M1 produced a numerically larger gap than M4 on average across the five *θ* points.

**Table 26:** Assembly performance by predicted-item share, averaged across Grades 3 through 12.

Predicted Share	Method	Mean TIF Gap	Constraint Violation Rate	Mean Ability RMSE	Mean Ability RMSE at θ=±2
0.20	M1	0.209	0.025	0.501	0.632
0.20	M4	0.128	0.025	0.506	0.610
0.40	M1	0.397	0.025	0.516	0.665
0.40	M4	0.198	0.025	0.514	0.631
0.60	M1	0.612	0.038	0.535	0.704
0.60	M4	0.276	0.025	0.522	0.647
0.80	M1	0.844	0.050	0.561	0.751
0.80	M4	0.361	0.038	0.535	0.666

## Data Availability

The data presented in this study are available on request from the corresponding author due to the empirical data is protected under state law.

## Supplementary Materials

The following supporting information can be downloaded at: https://www.mdpi.com/article/10.3390/jintelligence14090216/s1, Table S1: Transformer training hyperparameters; Table S2: Simulated annealing solver hyperparameters; Table S3: Early stopping behavior by model; Table S4: Uncertainty method comparison on the validation set; Table S5: Mean total test information at increasing time budgets, pooled across all bank sizes, error magnitudes, and test lengths. Algorithm S1: Penalty calibration loop. Algorithm S2: Real-time assembly loop.

## Abbreviations

The following abbreviations are used in this manuscript:

3PL	Three-parameter logistic model, the item response theory model used
2PL	Two-parameter logistic model, the item response theory model used
AI	Artificial Intelligence
BERT	Bidirectional Encoder Representations from Transformers
CAT	Computerized adaptive testing
IRT	Item response theory
K–12	Kindergarten through grade 12
LLMs	Large language models
MIP	Mixed-integer programming
NLP	Natural language processing
QUBO	Quadratic Unconstrained Binary Optimization
RMSE	Root mean square error
RoBERTa	A Robustly Optimized BERT Pretraining Approach, the encoder used
SD	Standard deviation
SEM	Standard error of measurement
TIF	Test information function
M1	Naive MIP, treats predicted parameters as exact
M2	Chance-constrained MIP
M3	Robust MIP, the budget-of-uncertainty formulation
M4	Mean–variance QUBO, also written MV QUBO, a proposed method
M5	Worst-case QUBO, also written WC QUBO, a proposed method
M6	Greedy heuristic
MV	Mean–variance, the objective used by method M4
WC	Worst-case, the objective used by method M5
