# Supplementary material for: Real-Time Dynamic Adaptive Test Assembly for Personalized Measurement of Cognitive Abilities Under Continuous Item Bank Growth
Source: J Intell. 2026 Sep 6;14(9):216. doi: 10.3390/jintelligence14090216 (PMC13608447; doi:10.3390/jintelligence14090216)
Supplement: Supplementary file 1 [file jintelligence-14-00216-s001.zip › jintelligence-4473879-supplementary.pdf]

Supplementary File

S1. Transformer training hyperparameters

The pooled Classify token ([CLS]) representation produced by the encoder is passed through a two-layer feedforward regression layer with hidden dimension 256, Gaussian Error Linear Unit (GELU) activation, and dropout rate 0.10. The [CLS] is a special placeholder symbol prepended to the input. After the encoder processes the whole sequence, the vector at the [CLS] position serves as a fixed-length numerical summary of the entire item text, similar to how a factor score summarizes a set of indicators. The feedforward regression head is a small fully connected neural network that maps this summary vector to the predicted item parameters. The GELU activation is the nonlinear function applied between the layers of this small network, in the same role that the logistic function plays inside a generalized linear model. The dropout rate of 0.10 means that during training a random ten percent of the connections in the head are temporarily set to zero on each pass, which is a regularization technique that prevents the network from overfitting the small set of calibrated items. The output layer has four units producing the predictive mean of log discrimination, the predictive mean of difficulty, and the log of the predictive variances for each parameter. The log variance parameterization ensures positive predicted variances without an explicit constraint. Table S1 lists the hyperparameters used to train the transformer prediction model described in Section 2.2.

Table S1. Transformer training hyperparameters.

Hyperparameter	Value
Base model	RoBERTa-base, 125M parameters
Max sequence length	512 tokens
Batch size	16
Learning rate	2e-5 with linear warmup
Warmup proportion	0.1
Weight decay	0.01
Optimizer	AdamW
Maximum epochs	20
Early stopping patience	3 epochs on validation NLL
Dropout rate	0.1
Regression head hidden dim	256
Ensemble members	5
Random seeds	42, 43, 44, 45, 46

S2. Propagating item-parameter uncertainty into the information function

This step yields, for every item and every target ability point, the two summary numbers that the mean variance objective requires in closed form. For predicted item i, the predictive distribution on $(\log a_i, b_i)$ has mean $\hat{\mu}_i$ and covariance $\hat{\Sigma}_i$. To propagate this uncertainty into the information function $I_i(\theta)$ at a target ability θ_t, the delta method approximates the distribution of a nonlinear function of a random vector by a normal distribution with mean equal to the function evaluated at the mean of the input vector and variance equal to the gradient squared norm under the input covariance.

Let $g_i(\log a, b) = \left(e^{\log a}\right)^2 P_i\left(\theta_t; e^{\log a}, b\right)\left[1 - P_i\left(\theta_t; e^{\log a}, b\right)\right]$; the expected information at θ_t is approximated by

$$\mu_i^{(t)} = g_i\left(\hat{\mu}_i^{\log a}, \hat{\mu}_i^{b}\right) \tag{S1}$$

and the variance of the information is approximated by

$$\tau_i^{(t)} = \nabla g_i^{\top}\,\hat{\Sigma}_i\,\nabla g_i \tag{S2}$$

where ∇g_i is evaluated at the predictive mean $\left(\hat{\mu}_i^{\log a}, \hat{\mu}_i^{b}\right)$. The gradient components, derived by direct differentiation of Equation (2), are

$$\frac{\partial g_i}{\partial \log a} = 2a_i^2 P_i(1-P_i) + a_i^2 P_i(1-P_i)(1-2P_i)\cdot a_i(\theta_t - b_i) \tag{S3}$$

$$\frac{\partial g_i}{\partial b} = -a_i^3 P_i(1-P_i)(1-2P_i) \tag{S4}$$

with $a_i = e^{\log a}$ and $P_i = P_i(\theta_t)$ evaluated at the predictive mean. For calibrated items the information variance is obtained by propagating the marginal maximum likelihood standard errors of the discrimination and difficulty parameters through the same delta method used for predicted items, so that calibrated items carry nonzero variance in the assembly objective. Table 16 in the main text reports the sensitivity of the assembly results to this choice by comparing the two treatments. As a check on the first order delta method, a Monte Carlo alternative draws 500 samples from a bivariate normal $\mathcal{N}\left(\hat{\mu}_i, \hat{\Sigma}_i\right)$, computes the information at each draw, and uses the empirical mean and variance. We use the delta method in the main analyses because it is differentiable and orders of magnitude faster, and we use Monte Carlo as a validation step on a random subsample of items.

S3. Penalty calibration procedure

For Equation (16), substituting Equations (9) through (15) and expanding all squares produces a sum of constant, linear, and pairwise terms in $\boldsymbol{x}$. This is the canonical QUBO form

$$H(\mathbf{x}) = \sum_{i=1}^{N'} Q_{ii}\,x_i + \sum_{i<j} Q_{ij}\,x_i x_j + \text{const} \tag{S5}$$

where N' includes the original items together with all slack variables, and Q is the QUBO coefficient matrix. The Ising mapping uses the substitution

$$x_i = \frac{1 + s_i}{2}, \quad s_i \in \{-1, +1\} \tag{S6}$$

to convert the binary form into the spin form. The two formulations carry identical information about the optimization landscape, and the choice between them depends on which form the chosen solver accepts. The simulated annealing implementation we use accepts the QUBO form natively.

The penalty coefficients $P_1, \ldots, P_5$ must be large enough that constraint violations dominate any objective gain from violation, and small enough that the objective landscape remains numerically tractable. We use an adaptive scheme that initializes each penalty at the largest possible objective contribution, runs the solver for a small number of runs, measures violation magnitudes, and inflates penalties multiplicatively for any constraint that remained violated. Convergence is declared when all violations are zero across five consecutive iterations, or when 50 iterations are reached, whichever comes first. Calibrated coefficients are then frozen and used for all subsequent instances of the same problem structure. This has been detailed in Algorithm S1 as follows, which describes the adaptive calibration loop used to set the penalty coefficients in the QUBO formulation. The goal is to find coefficients large enough that

constraint violations always outweigh any objective gain from violating them, while keeping the objective landscape numerically tractable.

Algorithm S1. Penalty calibration loop.

1. Initialize $P_j^{(0)} = \max_i \left|\mu_i^{(t)}\right| \cdot n$ for $j = 1, \dots, 5$.
2. For iteration $r = 0, 1, 2, \dots$ until convergence:
   - Construct $H^{(r)}$ with current penalties.
   - Solve via simulated annealing for $R = 50$, retain best feasible result if any, otherwise best result.
   - For each constraint type j, compute violation magnitude $v_j^{(r)}$.
   - Update $P_j^{(r+1)} = P_j^{(r)} \cdot \left(1 + \gamma v_j^{(r)}\right)$ with $\gamma = 0.5$ if violated, otherwise hold constant.

Convergence: all violations zero across 5 consecutive iterations or 50 iterations reached.

S4. Ising solver details

Simulated annealing is a probabilistic local search that proposes random bit flips and accepts them according to a temperature-dependent Metropolis criterion. That is, the solver starts from a random selection of items and at each step proposes to add an item that is currently out or to drop an item that is currently in. A flip that improves the objective is always accepted. A flip that worsens the objective is accepted with a probability that decreases as the worsening becomes larger and as the temperature parameter decreases. This conditional acceptance rule is known as the Metropolis criterion, and it allows the search to occasionally accept worse solutions, which is what enables it to escape local optima. The temperature is lowered on a schedule, so that early in the run the search behaves like a random walk that escapes local minima readily, and late in the run the search behaves like greedy descent. Within this process, a read is one complete annealing run from a random initialization down to the lowest temperature on the cooling schedule. Each read produces one candidate solution, and different reads can land at different local minima. A sweep is one pass through all of the binary variables in the QUBO, in which the solver visits each variable once and proposes a flip according to the Metropolis criterion. The number of reads controls how many independent runs the solver performs before returning the best energy solution across the ensemble. The number of sweeps within one read controls how thoroughly each individual run explores its local neighborhood. Total compute time grows approximately as the product of the number of reads, the number of sweeps per read, and the QUBO dimension.

Table S2 lists the values of these hyperparameters that we use throughout, with 200 reads fixed across all bank sizes and the sweep schedule grown proportionally with the QUBO dimension.

Table S2. Simulated annealing solver hyperparameters.

Hyperparameter	Selected Value
Algorithm	Simulated annealing
Implementation Sampler	D-wave-neal
Number of reads	200 (parallelized across CPU cores)
Sweeps per read	$10 \times N'$
Beta schedule	Geometric, 1000 steps
Beta range	$[\beta_{\min}, \beta_{\max}]$ auto-tuned per instance
Random seed	Documented per run
Time budget	1.0 second per instance for personalized loop

The solver returns up to 200 candidate solutions ranked by energy. The post processing step iterates through candidates from lowest to highest energy, returns the first one that satisfies all hard constraints, and records the energy gap and feasibility rate for diagnostics. Two additional Ising style solvers are used in sensitivity analyses. The first is parallel tempering via *D-wave-neal* with tempering, and the second is tabu search via *D-wave-tabu*. Parallel tempering is an extension of simulated annealing that runs several Markov chains in parallel at different fixed temperatures and occasionally swaps configurations between chains, which improves exploration of rugged objective landscapes. Tabu search is a different local search method that systematically forbids the solver from revisiting recently flipped variables for a set number of steps, which prevents the search from immediately undoing its own moves and forces it to explore new regions of the binary space. For real-time deployment in the personalized loop, warm starting from the previous solution accelerates resolves when the item bank grows incrementally. The previous solution $\boldsymbol{x}^{(t-1)}$ initializes a fraction of the reads, and the remaining reads use random initialization to maintain exploration.

S5. Assembly deployment workflow

The personalized assembly loop detailed in Algorithm S2 proceeds as follows. The system receives the current student state, which includes the current ability estimate θ, the items previously administered, and the target content standard. The item bank is updated by appending any newly authored items together with their transformer predicted parameters and uncertainty, and by retiring any over exposed items. Three target ability points are placed at $\theta - 0.5$, θ, and $\theta + 0.5$ with weights 0.25, 0.50, and 0.25, which produces a smooth information profile centered on the current ability estimate. The QUBO matrix is constructed from the current bank using Equations (9) through (15), and the QUBO is solved by simulated annealing with a one second time budget and warm start from the previous solution. The returned feasible solution is validated against all hard constraints and the selected items are returned to the student. Once responses are received, the ability estimate is updated by maximum likelihood or by expected a posteriori estimation, and the loop repeats. This workflow preserves the same QUBO interface across calibrated and predicted items, which means that no structural change to the assembly pipeline is required as items mature from predicted to calibrated.

Algorithm S2. Real-time assembly loop.

1. Receive student state: current ability estimate $\hat{\theta}$, prior items administered, target standard.
2. Update item bank: append any newly authored items with transformer-predicted parameters and uncertainty, retire over-exposed items.
3. Set target ability points: $\theta_1 = \hat{\theta} - 0.5$, $\theta_2 = \hat{\theta}$, $\theta_3 = \hat{\theta} + 0.5$, with weights $(0.25, 0.5, 0.25)$.
4. Construct QUBO matrix Q from current bank.
5. Solve QUBO via simulated annealing with time budget 1.0 second and warm start from previous solution.
6. Validate the returned feasible solution satisfies all hard constraints.
7. Return selected items to student.
8. Receive responses, update $\hat{\theta}$ via maximum likelihood or expected a posteriori.
9. Return to step 1.

S6. Validation

Table S3 records the epoch at which validation loss reached its minimum together with the epoch at which early stopping halted training. Validation loss reaches its minimum at epoch 5.0 on average against a maximum of twenty epochs, and the gap between training and validation loss at that point is 0.141. This pattern indicates validation performance plateaus after only a few epochs and the train-validation gap then grows, consistent with rapid overfitting in the smaller samples and with the frozen-encoder model's comparable accuracy. Table S4 reports the uncertainty method comparison, and Table S5 reports pooled anytime solution quality, which lists the mean runtime in seconds by method and bank size, averaged across error magnitudes and test lengths.

Table S3. Early stopping behavior by model.

Model	Min val NLL epoch	Stop epoch	Min val NLL	Train NLL
Grade 3	4	7	1.286	1.109
Grade 4	5	8	1.241	1.083
Grades 5–12	4	7	1.318	1.122
Cross-grade	6	9	1.104	1.012
Pooled	6	9	1.087	1.006

Table S4. Uncertainty method comparison on the validation set.

Method	RMSE $log\ a$	RMSE b	Cov 90% $log\ a$	Cov 90% b	Cost/item
Deep ensemble, 5 members	0.512	1.061	0.875	0.850	58.4 ms
MC dropout, 50 passes	0.528	1.094	0.800	0.725	486.7 ms

Table S5. Mean total test information at increasing time budgets, pooled across all bank sizes, error magnitudes, and test lengths.

Method	0.1 s	0.5 s	1.0 s	2.0 s	5.0 s
M4. MV QUBO	6.18	6.69	7.69	7.95	8.11
M5. WC QUBO	6.41	6.94	7.71	7.71	8.07
